# A mathematical model to serve as a clinical tool for assessing obstructive sleep apnea severity

**DOI:** 10.3389/fphys.2023.1198132

**Published:** 2023-08-03

**Authors:** Nida T. Qayyum, C. Hunter Wallace, Rami N. Khayat, Anna Grosberg

**Affiliations:** ^1^ Department of Chemical and Biomolecular Engineering, University of California, Irvine, Irvine, CA, United States; ^2^ UCI Edwards Lifesciences Foundation Cardiovascular Innovation and Research Center (CIRC), University of California, Irvine, Irvine, CA, United States; ^3^ Department of Biomedical Engineering, University of California, Irvine, Irvine, CA, United States; ^4^ The UCI Sleep Disorders Center, University of California, Irvine, Irvine, CA, United States; ^5^ Center for Complex Biological Systems, University of California, Irvine, Irvine, CA, United States; ^6^ NSF-Simons Center for Multiscale Cell Fate Research, University of California, Irvine, Irvine, CA, United States; ^7^ Sue and Bill Gross Stem Cell Research, University of California, Irvine, Irvine, CA, United States

**Keywords:** desaturation, hypoxia, hypoxemia, breathing, oxygenation, hypopnea, mass transfer

## Abstract

Obstructive sleep apnea (OSA) is a sleep disorder caused by periodic airway obstructions and has been associated with numerous health consequences, which are thought to result from tissue hypoxia. However, challenges in the direct measurement of tissue-level oxygenation make it difficult to analyze the hypoxia exposure pattern in patients. Furthermore, current clinical practice relies on the apnea-hypopnea index (AHI) and pulse oximetry to assess OSA severity, both of which have limitations. To overcome this, we developed a clinically deployable mathematical model, which outputs tissue-level oxygenation. The model incorporates spatial pulmonary oxygen uptake, considers dissolved oxygen, and can use time-dependent patient inputs. It was applied to explore a series of breathing patterns that are clinically differentiated. Supporting previous studies, the result of this analysis indicated that the AHI is an unreliable indicator of hypoxia burden. As a proof of principle, polysomnography data from two patients was analyzed with this model. The model showed greater sensitivity to breathing in comparison with pulse oximetry and provided systemic venous oxygenation, which is absent from clinical measurements. In addition, the dissolved oxygen output was used to calculate hypoxia burden scores for each patient and compared to the clinical assessment, highlighting the importance of event length and cumulative impact of obstructions. Furthermore, an intra-patient statistical analysis was used to underscore the significance of closely occurring obstructive events and to highlight the utility of the model for quantitative data processing. Looking ahead, our model can be used with polysomnography data to predict hypoxic burden on the tissues and help guide patient treatment decisions.

## 1 Introduction

Obstructive sleep apnea (OSA) is a sleep-related breathing disorder caused by repeated pharyngeal collapse, which leads to episodes of restricted breathing (hypopnea) or ceased breathing (apnea) (Shah et al., 2021). Consequently, if left untreated, OSA hasbeen linked to health problems including hypertension, worsening cardiovascular disease outcomes, metabolic disorders, and cognitive dysfunction (Lam et al., 2006; Bucks et al., 2013; Kendzerska et al., 2014; Wu et al., 2016). Qualitatively, an increasing severity of OSA is believed to be associated with a higher risk of developing consequences such as hypertension (Marin et al., 2012). Currently, OSA severity is primarily measured using the apnea-hypopnea index (AHI), which has not been shown as a reliable predictor of disease progression in patients with comorbid cardiovascular disease (Kendzerska et al., 2014; Wu et al., 2016). In reality, a better assessment of pathological effects requires a mechanistic understanding of tissue exposure to hypoxia and its impact on various target tissues and organs. Furthermore, considering the range of OSA severity, it is also important to conduct patient-specific studies for a more accurate quantification of health risks.

The hypoxia exposure pattern in OSA is the most important pathophysiologic abnormality accounting for the cardiovascular consequences of OSA. Direct measurement of the blood oxygen concentration at the level of the deeper tissues is difficult to achieve non-invasively, and pulse oximetry is not sufficiently accurate (Mardirossian and Schneider, 1992). Considering this, mathematical modeling could potentially bridge the gap between available clinical data and quantification of OSA impact on tissue function. Therefore, the challenge is to develop a model, which outputs blood oxygen concentration at the tissue level using input breathing data. Previous models of sleep-related breathing disorders have been developed to understand the resulting physiological impacts (Cheng et al., 2010; Cheng and Khoo, 2012). Although these representations are useful for a general understanding of hemoglobin desaturation during OSA, it would be of greater clinical relevance to incorporate patient data for a more individualized assessment. For optimal clinical relevance, the ideal model would be one that incorporates data collected from polysomnography, the standard diagnostic test for OSA. Yet, this approach has not been thoroughly explored in current OSA literature. In addition, many models are complex, with some requiring hundreds of parameters (Cheng et al., 2010; Cheng and Khoo, 2012). Accordingly, it would be of great value to develop a model that can be easily deployed within a clinical polysomnography to generate a quantitative assessment of OSA severity by directly modeling tissue intermittent hypoxia exposure.

Therefore, we aimed to develop a clinically deployable mathematical model, with few parameters and the capability of taking time-dependent breathing and heart rate data as inputs, to simulate the blood oxygen concentration at the level of the deeper tissues. Simulations of a normal breathing pattern and severe OSA were used to validate the model. Additional simulated breathing patterns with events of apneas/hypopneas were also used to study cases of varying OSA severity, different ventilatory responses, the implications of unscored obstructive events, and the effect of apnea/hypopnea duration. Furthermore, to demonstrate possible clinical applications, respiratory and heart rate data from two OSA patients was used as an input to the model. In combination, the results illustrate the future clinical value of the model in assessing tissue-level hypoxia exposure patterns.

## 2 Methods and model formulation

The model was built using analytical equations that were solved in MATLAB R2022b. Details of all model variables and physiological and fitting parameters are provided in Supplementary Tables S1–S4. The physiological parameters were taken from or calculated using literature values. Furthermore, for each OSA patient, a fitting parameter was used to convert nasal pressure data into a time-dependent lung volume. Essential definitions of variables and parameters are provided in Tables 1, 2, respectively. Additional details of model derivation and MATLAB implementation are provided in the Supplementary Material for anyone who may be interested in reproducing the model.

**TABLE 1:** Definitions of model variables and terms.

Variable	Definition
$C_{A,O_{2}}$	Dissolved oxygen concentration in alveolar membrane
$C_{c,O_{2}}$	Spatially averaged dissolved oxygen concentration in pulmonary capillary compartment
*C*_*cv*,*i*_	Oxygen concentration in control volume: i = d (dissolved), T (total)
$C_{j,O_{2}i}$	Oxygen concentration: j = pa (pulmonary arteries), pc (pulmonary capillary compartment), pv (pulmonary veins), sa (systemic arteries), sv (systemic veins) and i = d (dissolved), T (total)
$P_{A,O_{2}}$	Alveolar oxygen partial pressure
$P_{j,O_{2}}$	Oxygen partial pressure (j as defined for $C_{j,O_{2}i}$ )
$S_{j,O_{2}}$	Fraction of hemoglobin oxygen saturation (j as defined for $C_{j,O_{2}i}$ )
*t*	Time
ΔV	Differential volume
*V*_*A*_	Simulated alveolar volume
*z*	Spatial coordinate

**TABLE 2:** Definitions of physiological parameters.

Parameter	Definition
*A*_*eff*_	Effective cross-sectional area of all pulmonary capillaries
*β*_*p*_	Oxygen solubility in alveolar-capillary membrane and blood plasma
*b*_*r*_	Breathing rate
*C*_*Hb*_	Hemoglobin concentration in blood
$D_{L,O_{2}}$	Oxygen lung diffusing capacity
*k*_*l*_	Lung mass transfer coefficient
*k*_*pc*_	Pulmonary capillary control volume mass transfer coefficient
*L*_*c*_	Pulmonary capillary compartment length
${MR}_{O_{2}}$	Total basal metabolic rate for oxygen consumption
*P*_*B*_	Barometric pressure
$P_{H_{2}O}$	Water vapor pressure at normal body temperature
$P_{I,O_{2}}$	Inspired oxygen partial pressure
*R*	Ideal gas constant
*T*	Normal body temperature
*V*_*D*_	Dead space volume
*V*_*End*_	End-expiration alveolar volume
*V*_*pc*_	Total pulmonary capillaries blood volume
*V*_*sys,cap*_	Total systemic capillaries blood volume
*V*_*T*_	Tidal volume
*V*_*vent*_	Alveolar ventilation volume
$y_{O_{2}}$	Inspired oxygen mole fraction

### 2.1 Pulmonary arterial oxygenation

The systemic capillaries were modeled as a single compartment (Supplementary Figure S1). All oxygen transfer to the tissues in the systemic circulation was assumed to occur in the capillary region. This was parameterized using an overall metabolic rate for tissue oxygen consumption. Therefore, the total oxygen concentration in the pulmonary arteries was taken to be equivalent to that in the systemic veins $\left(C_{pa,O_{2}T}\approx C_{sv,O_{2}T}\right)$ . Similarly, the total oxygen concentration in the pulmonary veins was taken to be equivalent to that in the systemic arteries $\left(C_{pv,O_{2}T}\approx C_{sa,O_{2}T}\right)$ , assuming that transfer out of the arterioles is not significant. If necessary, oxygen leakage in the arteriole system can be incorporated by equating the total oxygen concentration entering the systemic capillary compartment to a fraction of that leaving the pulmonary capillary compartment $\left(C_{sa,O_{2}T}=fraction\cdot C_{pv,O_{2}T}\right)$ .

To determine the total oxygen concentration in the pulmonary arteries, an unsteady-state mass balance was performed over a differential control volume moving through the systemic capillary compartment at the same velocity as the surrounding blood:

$$\frac{dC_{cv,T}}{dt}=-\frac{{MR}_{O_{2}}}{V_{\mathrm{sys,cap}}}$$ (1)

Eq. 1 was solved over time to determine the relation between the total oxygen concentration in the pulmonary arteries and veins (Supplementary Appendix S1.1).

An important point to highlight is that the total concentration of oxygen in the blood was taken as the sum of the dissolved and hemoglobin-bound components. The dissolved oxygen concentration is necessary to consider as it drives the gradients for mass transfer, and it is what the tissues are exposed to. In other words, oxygen must dissociate from hemoglobin and dissolve into the blood plasma before it can be transferred to the body tissues.

$$C_{j,O_{2}T}\left(t\right)=C_{j,O_{2}d}\left(t\right)+4\cdot C_{Hb}\cdot S_{j,O_{2}}\left(t\right)$$ (2)

In Eq. 2, *j* = *pa*, *pc*, *pv*, *sa*, and *sv* for the pulmonary arteries, pulmonary capillary compartment, pulmonary veins, systemic arteries, and systemic veins, respectively.

The relation between hemoglobin saturation and oxygen partial pressure was estimated using a fit to the standard oxygen-hemoglobin dissociation curve (Severinghaus, 1979):

$$S_{j,O_{2}}\left(t\right)={\left(1+\frac{\left(23,400\;{mmHg}^{3}\right)}{{\left(P_{j,O_{2}}\left(t\right)\right)}^{3}+\left(150\;{mmHg}^{2}\right)\;P_{j,O_{2}}\left(t\right)}\right)}^{-1}$$ (3)

$$P_{j,O_{2}}\left(t\right)=\frac{C_{j,O_{2}d}\left(t\right)}{\beta_{p}}$$ (3a)

In Eqs 3, 3a, j is the same as defined in Eq. 2.

### 2.2 Pulmonary capillary mass transfer

The pulmonary capillaries were modeled as a single compartment, assuming no significant regional heterogeneity in lung ventilation/perfusion (Supplementary Figure S2). An unsteady-state mass balance was performed over a control volume moving within the compartment to obtain a time-dependent, spatial oxygen profile:

$$\Delta V\frac{dC_{cv,T}}{dt}=k_{pc}\left(C_{A,O_{2}}\left(t\right)-C_{cv,d}\left(t\right)\right)$$ (4)

$$k_{pc}=\frac{D_{L,O_{2}}}{\beta_{p}}\left(\frac{\Delta z}{L_{c}}\right)$$ (4a)

$$L_{c}=\frac{V_{pc}}{A_{\mathrm{eff}}}$$ (4b)

$$C_{A,O_{2}}\left(t\right)=\beta_{p}\cdot P_{A,O_{2}}\left(t\right)$$ (4c)

The total oxygen concentration within the control volume was assumed to be spatially uniform. In addition, the mass transfer coefficient was assumed to be uniform along the length of the pulmonary capillary compartment. Pulmonary blood velocity was used to track the movement of the control volume along the pulmonary capillaries and to allow for the effect of perfusion on the mass transfer mechanism (more details provided in Supplementary Appendix S1.2). In addition, the effect of the *O*_2_ − *Hb* reaction was incorporated by using Eqs 2, 2 3, to calculate the dissolved oxygen concentration. The oxygen mass transfer rate was determined using the molar flux across the alveolar-capillary membrane. Pseudo-steady state was assumed to be valid for this transfer process as the diffusion time was much smaller than the time for boundary changes (Supplementary Appendix S3). The dissolved oxygen in the alveolar membrane was assumed to be uniform along the length of the pulmonary capillary compartment. Furthermore, the solubility factor for oxygen was assumed to be the same for the membrane space and blood plasma.

Since the plasma and red blood cells are not separately modeled, the individual diffusion and reaction coefficients (consisting of diffusion through the alveolar-capillary membrane, blood plasma, red blood cells, and facilitated diffusion due to the oxygen-hemoglobin interaction) are not known. Therefore, a physiologic estimate of the lung diffusive capacity was used, which inherently incorporates these terms and is around 21 mL *O*_2_ ⋅min^−1^ ⋅mmHg^−1^ for a normal subject at rest (Guyton and Hall, 2000). Data from a clinical paper showed that the average lung oxygen diffusive capacity did not significantly deviate from this normal value under the condition of anoxia (Fishman, 1954). Therefore, it was assumed to be constant for all cases (simulated and patient).

### 2.3 Alveolar mass transfer

The alveoli were modeled as a single, homogeneous compartment (Supplementary Figure S1). An unsteady-state mass balance was performed for the inspiration and expiration processes to determine the alveolar oxygen partial pressure in response to input breathing data. The inspired air was assumed to be ideal, saturated with water vapor, at normal body temperature, and at a 0.21 oxygen mole fraction. With no spatial variations in gas concentration, the oxygen partial pressure leaving the alveolar compartment was taken to be equivalent to that within it. Mass transfer out of the alveolar compartment was calculated using the concentration gradient in dissolved oxygen across the alveolar-capillary membrane. A spatially averaged oxygen concentration along the pulmonary capillary compartment $\left(C_{c,O_{2}}\right)$ was used for this gradient. The resulting differential equations are similar to those presented in previous literature (Reynolds et al., 2010).

During inspiration, when $\frac{dV_{A}}{dt}>0$ , the alveolar partial pressure was determined using:

$$V_{A}\left(t\right)\frac{dP_{A,O_{2}}}{dt}=\frac{dV_{A}}{dt}\left(P_{I,O_{2}}-P_{A,O_{2}}\left(t\right)\right)-k_{l}\left(C_{A,O_{2}}\left(t\right)-C_{c,O_{2}}\left(t\right)\right)R\cdot T$$ (5)

$$P_{I,O_{2}}=y_{O_{2}}\left(P_{B}-P_{H_{2}O}\right)$$ (5a)

$$k_{l}=\frac{D_{L,O_{2}}}{\beta_{p}}$$ (5b)

$$C_{c,O_{2}}\left(t\right)=\frac{1}{L_{c}}\int_{0}^{L_{c}}C_{pc,O_{2}d}\left(z,t\right)\;dz$$ (5c)

During expiration, when $\frac{dV_{A}}{dt}<0$ , the alveolar partial pressure was determined using:

$$V_{A}\left(t\right)\frac{dP_{A,O_{2}}}{dt}=-k_{l}\left(C_{A,O_{2}}\left(t\right)-C_{c,O_{2}}\left(t\right)\right)R\cdot T$$ (6)

### 2.4 Normal and simulated OSA breathing patterns

An approximation of a normal breathing pattern was used to validate the model (Reynolds et al., 2010):

$$V_{A}\left(t\right)=\frac{1}{2}V_{\mathrm{vent}}\sin\left(2\pi \cdot b_{r}\cdot t-\frac{\pi }{2}\right)+\left(\frac{1}{2}V_{\mathrm{vent}}+V_{\mathrm{End}}\right)$$ (7)

$$V_{\mathrm{vent}}=V_{T}-V_{D}$$ (7a)

Variations of this breathing pattern were used to simulate multiple cases of OSA for assessing the AHI. This was done by varying the breathing rate and tidal volume and introducing apnea/hypopnea events. The AHI for each OSA simulation was defined as the number of apnea/hypopnea events divided by the total breathing time studied (in hours) after achieving model stability. All simulated breathing patterns represented the alveolar volume, and the duration of inspiration and expiration was assumed to be equivalent for each. In these simulations, a sinusoidal function was used to approximate breathing, but the model has the capability to use any form of input breathing data.

### 2.5 OSA patient breathing pattern

Nasal pressure data from two OSA patients was obtained during multi-hour sleep studies, which were conducted with and without continuous positive airway pressure (CPAP) administration. Human protection: the analysis was done using de-identified datasets generated from studies performed in the UCI Sleep Center. The research was done in compliance with the UCI IRB regulations (UCI IRB #267). For each patient, the recorded pressure during a portion of the study without CPAP was converted to a time-dependent lung volume for implementation as a model input. To achieve this, each pressure signal was first normalized to a mean of zero by subtracting a moving average (taken over 70-s intervals for Patient 1 and 50-s intervals for Patient 2) from the raw signal (Supplementary Appendix S4; Supplementary Figures S4, S5). Following this, for each patient, a portion of the signal identified as normal breathing by a clinician was isolated. The breathing rate, determined from the isolated signal, and tidal volume, approximated using the patient’s height-based ideal body weight, were used to simulate a normal breathing pattern and corresponding flow rate for each case (Supplementary Appendix S4; Supplementary Figures S6, S7). Considering that the body-mass index (BMI) falls within the overweight range for Patient 1 (25 ≤ BMI < 30) and the obese range for Patient 2 (BMI ≥30) (Table 3), average functional residual capacities (FRC) measured in overweight and obese subjects suspected of having OSA (Abdeyrim et al., 2015) were used as the end-expiration volumes in the simulated breathing patterns. To convert the recorded nasal pressure to a nasal flow rate, a fitting parameter for each patient was defined by assuming that the average maximum nasal pressure during the identified normal breathing corresponds to the maximum simulated inspiratory flow for ideal tidal breathing. The calculated fitting parameters represent total nasal conductance, effectively combining air density, areas of flow, kinematic heat ratio, and inlet pressure for a laminar flow relationship (Mansour et al., 2002; Mansour et al., 2003). The generated nasal flow signals were then integrated over time to achieve time-dependent lung volumes, which were used to approximate the patient alveolar volumes as model inputs (Supplementary Appendix S4).

**TABLE 3:** OSA patient characteristics.

Patient	Age (yr)	Sex	Height (m)	Weight (kg)	BMI (kg/m^2^)
1	55	M	1.78	84.1	26.6
2	49	M	1.85	105.7	30.7

### 2.6 Numerical solution and outputs

All differential equations were solved using the finite difference method (Supplementary Appendix S1). The initial time steps for the simulations and patient cases were chosen to ensure model stability, convergence, and computational efficiency (Supplementary Appendix S5.4). Furthermore, all breathing patterns were introduced after allowing the model to stabilize (t = 360 s). For the results, all presented dissolved oxygen concentrations are in units of *μ*mol/L of blood. In addition, percent decreases in hemoglobin oxygen saturation and dissolved oxygen concentration were calculated by comparing the average normal values to the minimum points during the period of study (Supplementary Appendix S6.2). To determine the percent reduction in the average mass transfer to the tissues, the difference in systemic arterial and venous dissolved oxygen concentrations was averaged over the breathing pattern time and compared to the normal value (Supplementary Appendix S6.2).

### 2.7 Analysis of patient data and statistics

For analysis of the clinical performance of the model, each patient was given a proposed hypoxia burden score for a selected event series (Figure 8 for Patient 1 and Figure 9 for Patient 2) and for the entire period of study. To determine the scores, an average and standard deviation of the dissolved oxygen in the systemic arteries across multiple wake sequences were first calculated for both patients (Supplementary Appendix S6.3). A value for the area between the arterial dissolved oxygen curve for the sequence of analysis and the average arterial dissolved oxygen during wakefulness was calculated and normalized by the time of the analyzed sequence to give the hypoxia burden score. Furthermore, an individual burden score was determined for the hypoxia period over the full analysis sequence for each patient. The hypoxia state was defined as a value of the systemic arterial dissolved oxygen lower than one standard deviation below the average arterial dissolved oxygen during wakefulness. The area deviation for this hypoxia sequence was determined as shown in Supplementary Figure S10 and normalized by the total time spent in hypoxia. When assessing the scores, a higher value indicates more severe hypoxia burden. Using the average dissolved oxygen for wakefulness as the threshold to calculate area deviations, the hypoxia burden score for wakefulness would be approximately equal to 0. All equations and further details of the procedure are provided in Supplementary Appendix S6.3.

Furthermore, a statistical analysis was performed on data from Patient 1 to assess the significance of closely occurring obstructive events and to demonstrate the utility of the model for quantitative data processing. The variables studied were the systemic arterial and venous saturations and the difference in dissolved oxygen concentration across the systemic capillaries. Four separate intervals of time were selected, with each one including two obstructive events (apnea or hypopnea) and the subsequent desaturation periods identified in the clinic. An average of each variable was calculated for all four intervals (Supplementary Appendix S6.5). Interval duration was kept constant at 125 s. Overall averages of each variable, taken over the duration of the entire study, were subtracted from the interval averages to create the data set for analysis (Supplementary Appendix S6.5). *p*-values were computed using the one-sample *t*-test in MATLAB (“ttest”). The test assesses the hypothesis that the data comes from a distribution with a mean of zero. The changes in the variables due to obstructive events were determined to be significant at *p*-values lower than 0.05.

## 3 Results

### 3.1 Simulated normal subject

**FIGURE 1:**
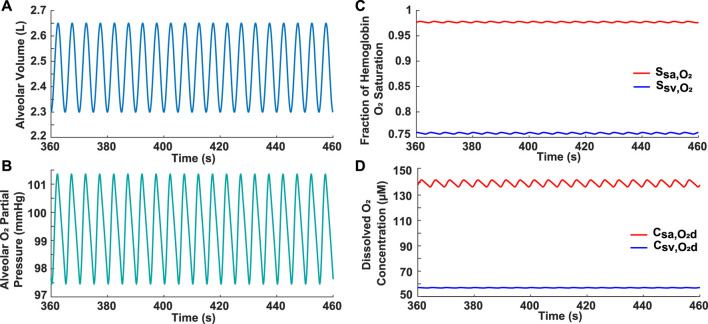
Results for normal subject. **(A)** Input simulated normal breathing pattern with *V*_*End*_ = 2.3 L, *V*_*T*_ = 0.5 L, *V*_*D*_ = 0.15 L, and *b*_*r*_ = 12 breaths/min. **(B)** Alveolar oxygen partial pressure. **(C)** Systemic arterial and venous hemoglobin oxygen saturation. **(D)** Dissolved oxygen concentration in systemic arteries and veins.

The model was first validated by comparing the results for a simulated normal breathing pattern at rest (Figure 1A) to expected physiological values. The average alveolar oxygen partial pressure was around 99 mmHg (Figure 1B). This is within the range of 98–104 mmHg stated in previous literature (Gardner, 1994; Guyton and Hall, 2000). The fraction of hemoglobin oxygen saturation was predicted to be around 0.98 in the systemic arteries and 0.76 in the systemic veins (Figure 1C), which approximates normal values of 0.97 and 0.75, respectively (Guyton and Hall, 2000). Furthermore, on average, the simulated dissolved oxygen concentration was 139 *μ*M in the systemic arteries and 57 *μ*M in the systemic veins (Figure 1D). When converted to oxygen partial pressures ( $P_{sa,O_{2}}$ = 99 mmHg and $P_{sv,O_{2}}$ = 41 mmHg), the values fall within expected ranges of ≈ 85–100 mmHg and ≈ 27–45 mmHg, respectively (Guyton and Hall, 2000; van Faassen et al., 2009; Ortiz-Prado et al., 2019).

### 3.2 Simulated severe OSA

**FIGURE 2:**
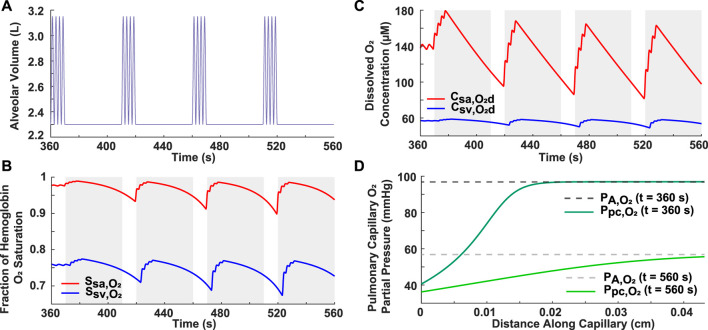
Analysis of simulated case of severe OSA. **(A)** Input breathing pattern with four 40-s apneas over a 3.3-min period, resulting in an AHI of 72. Hyperventilation is characterized by *V*_*T*_ = 1.0 L and *b*_*r*_ = 24 breaths/min. **(B)** Systemic arterial and venous hemoglobin oxygen saturation. **(C)** Dissolved oxygen concentration in systemic arteries and veins. The grayed portions indicate periods of ceased breathing **(B,C)**. **(D)** Alveolar and pulmonary capillary oxygen partial pressures for normal condition (t = 360 s) and at the end of the breathing pattern (t = 560 s).

A severe OSA breathing pattern was simulated to further validate the model and to assess the effect of frequent apneas on oxygenation in the systemic arteries and veins (Figure 2). The simulated pattern consisted of four 40-s apneas within a 3.3-min period (AHI = 72) (Figure 2A), which meets the severe OSA criteria of AHI ≥30. These obstructive events were separated by 10-s periods of hyperventilation (*V*_*T*_ = 1.0 L and *b*_*r*_ = 24 breaths/min) (Figure 2A). The resulting breathing pattern is similar to that in previous literature (Netzer et al., 2001; Cheng and Khoo, 2012). Realistically, this pattern would not be repeated multiple times over an hour as a patient would awaken once the hemoglobin saturation reached critically low values. Importantly, the model predicted a progressive decrease in the minimum hemoglobin oxygen saturation and dissolved oxygen concentration with each apnea (Figures 2B, C). For example, in the systemic arteries, the saturation fraction was around 0.93 after the first apnea, dropped to 0.91 after the second one, and further decreased to around 0.90 after the third apnea (Figure 2B). Another significant result is that the alveolar and end-pulmonary capillary oxygen partial pressures were not equivalent at the end of the studied breathing period (Figure 2D). Furthermore, it was noted that there is a brief time-delay from when each apnea starts to when the drop in oxygenation is felt in the systemic arteries and veins and, by extension, in the body tissues (Figures 2B, C).

### 3.3 Effect of ventilatory response on reoxygenation

**FIGURE 3:**
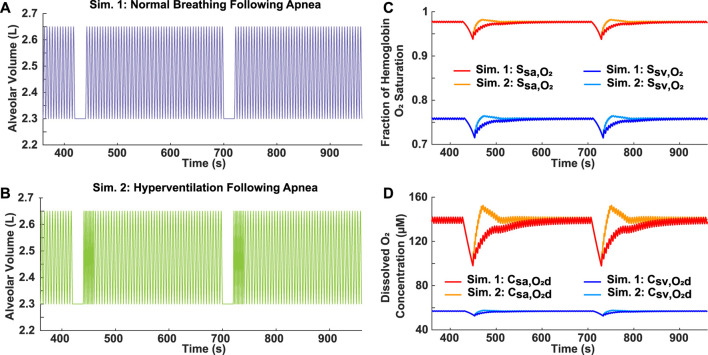
Analysis of reoxygenation following different ventilatory responses. **(A)** Simulation 1: Mild OSA breathing pattern (AHI = 12) with normal breathing following 20-s apnea. **(B)** Simulation 2: Mild OSA breathing pattern (AHI = 12) with 20-s period of hyperventilation (*b*_*r*_ = 24 breaths/min) following 20-s apnea, before resumption of normal breathing. **(C)** Systemic arterial and venous hemoglobin oxygen saturation for both simulations. **(D)** Dissolved oxygen concentration in systemic arteries and veins for both simulations.

Two variations of ventilatory response following an apnea were simulated to assess differences in oxygen recovery. In Simulation 1, the hypothetical patient resumed normal breathing (*b*_*r*_ = 12 breaths/min) following each 20-s apnea (Figure 3A). In Simulation 2, there was a 20-s period of hyperventilation (*b*_*r*_ = 24 breaths/min) after each apnea, followed by a resumption of normal breathing (Figure 3B). Considering that these breathing patterns incorporate two 20-s apneas over a 10-min period (AHI = 12), they could represent a case of mild OSA, which has a criteria of 5 ≤ AHI < 15. Based on these simulations, reoxygenation occurred faster when hyperventilation was initiated after an obstructive event. It took approximately 3.5 min, following each drop, to return to the normal saturation and dissolved oxygen concentration with a normal breathing response (Figures 3C, D). However, this time was reduced to around 0.5 min with hyperventilation included (Figures 3C, D). The drops were identical in both scenarios, but the hemoglobin oxygen saturations and dissolved oxygen concentrations increased past the predicted normal values for a short period during the hyperventilatory response.

### 3.4 Contribution of unscored obstructive events

#### 3.4.1 Unscored apneas

**FIGURE 4:**
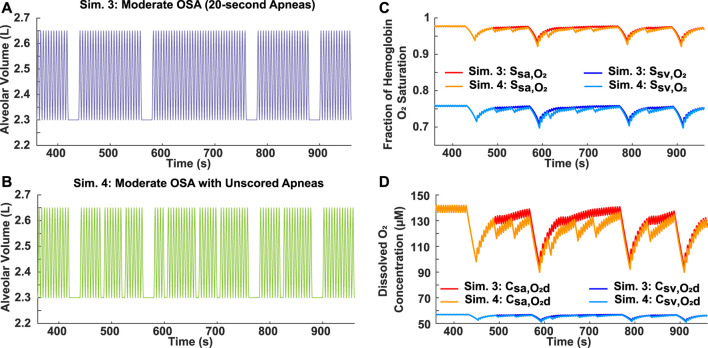
Analysis of AHI scoring criteria for apneas using moderate OSA breathing patterns. **(A)** Simulation 3: Input breathing pattern with four 20-s apneas over a 10-min period (AHI = 24) and no unscored obstructive events. **(B)** Simulation 4: Input breathing pattern with four 20-s apneas over a 10-min period (AHI = 24) and six unscored obstructive events, each with a 5-s duration. **(C)** Systemic arterial and venous hemoglobin oxygen saturation for both simulations. **(D)** Dissolved oxygen concentration in systemic arteries and veins for both simulations.

Two variations of moderate OSA breathing patterns were simulated to assess the scoring criteria of the AHI, which requires apneas to be at least 10 s long. In Simulation 3, four 20-s apneas were incorporated into a 10-min breathing period (Figure 4A). For Simulation 4, in addition to the four 20-s apneas, six 5-s apneas were also included (Figure 4B). Since the AHI does not account for the obstructive events lasting 5 s, both breathing patterns had the same score of 24, which is within the moderate OSA range of 15 ≤ AHI < 30. Although both cases have the same AHI, the breathing pattern with the unscored obstructive events caused a more severe oxygen deficiency (Figures 4C, D).

#### 3.4.2 Unscored hypopneas

**FIGURE 5:**
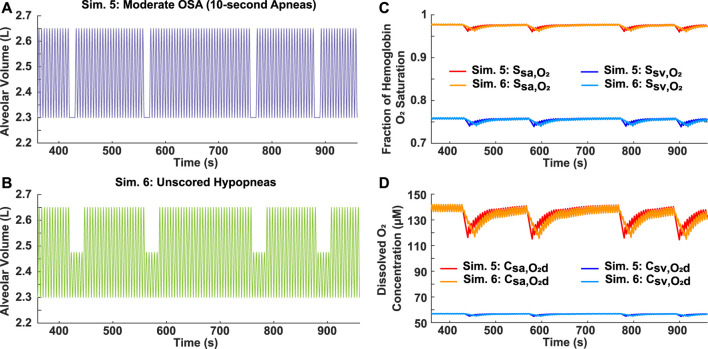
Analysis of AHI scoring criteria for hypopneas. **(A)** Simulation 5: Input moderate OSA breathing pattern with four 10-s apneas over a 10-min period (AHI = 24). **(B)** Simulation 6: Input breathing pattern with four 25-s unscored hypopneas over a 10-min period (AHI = 0), each with a 50% reduction in airflow. **(C)** Systemic arterial and venous hemoglobin oxygen saturation for both simulations. **(D)** Dissolved oxygen concentration in systemic arteries and veins for both simulations.

The scoring criteria of the AHI for apneas and hypopneas differs. Unlike an apnea, which must last longer than 10 s, a hypopnea also needs achieve at least a 4% decrease in oxygen saturation to be considered as an obstructive event. Therefore, to assess the importance of hypopneas in evaluating the effects of OSA, two differing breathing patterns were compared. In Simulation 5, four 10-s apneas were incorporated into a 10-min period, resulting in a moderate AHI of 24 (Figure 5A). For Simulation 6, four 25-s hypopneas, each with a 50% airflow reduction, were incorporated into a 10-min period (Figure 5B). However, since the decrease for the hemoglobin oxygen saturation in the systemic arteries and veins was less than 4% (Supplementary Table S5), the AHI for Simulation 6 is 0. Interestingly though, the results showed very similar saturation and dissolved oxygen concentration profiles for both scenarios (Figures 5C, D).

### 3.5 Significance of individual apnea duration

#### 3.5.1 Variable AHI

**FIGURE 6:**
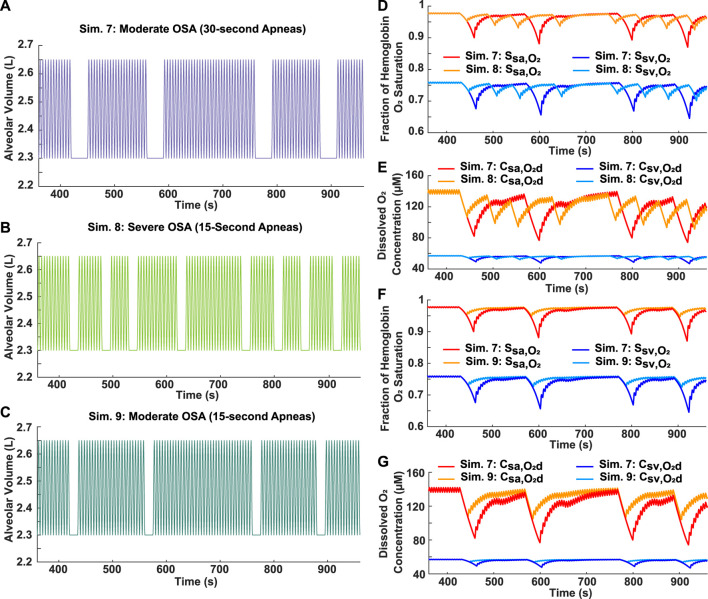
Analysis of apnea frequency and duration. **(A)** Simulation 7: Input moderate OSA breathing pattern with four 30-s apneas over a 10-min period (AHI = 24). **(B)** Simulation 8: Input severe OSA breathing pattern with eight 15-s apneas over a 10-min period (AHI = 48). **(C)** Simulation 9: Input breathing pattern with four 15-s apneas over a 10-min period (AHI = 24). **(D)** Systemic arterial and venous hemoglobin oxygen saturation for Simulations 7 and 8. **(E)** Dissolved oxygen concentration in systemic arteries and veins for Simulations 7 and 8. **(F)** Systemic arterial and venous hemoglobin oxygen saturation for Simulations 7 and 9. **(G)** Dissolved oxygen concentration in systemic arteries and veins for Simulations 7 and 9.

Two breathing patterns with the same total time of obstructed breathing (2 min), but varying AHI scores, were simulated to assess the relative importance of individual apnea duration and frequency in determining the severity of decrease in blood oxygen levels. For Simulation 7, four 30-s apneas were incorporated into a 10-min breathing pattern, resulting in a moderate AHI of 24 (Figure 6A). In Simulation 8, eight 15-s apneas were incorporated into a 10-min breathing pattern, resulting in a severe AHI of 48 (Figure 6B). Although the case with the higher AHI had more oscillations in oxygen levels, the degree of hypoxemia was more severe for the case with the longer individual apnea duration (Figures 6D, E).

#### 3.5.2 Constant AHI

Two moderate OSA breathing patterns with an AHI score of 24 were simulated to assess the relation between individual apnea duration and the severity of decrease in blood oxygen levels. Individual apnea lengths of 15 and 30 s were tested (Figures 6A, C, respectively). The model predicted more severe drops in oxygen levels for the longer apnea duration (Figures 6F, G). In addition, the time required for reoxygenation appeared to be longer as the apnea duration increased (Figures 6F, G).

### 3.6 Relative importance of AHI and apnea duration in assessing OSA severity

**FIGURE 7:**
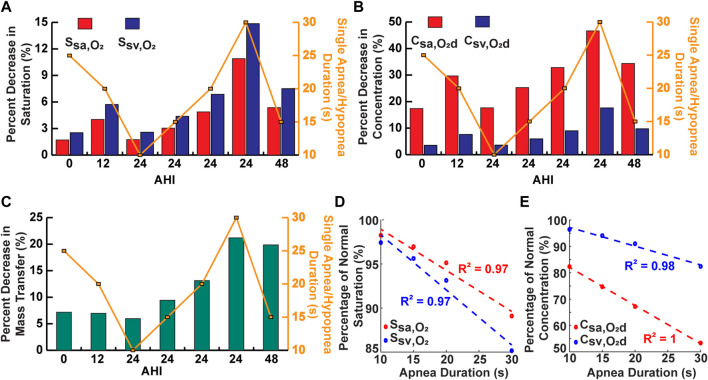
Analysis of single apnea/hypopnea duration and AHI. **(A)** Comparison of some simulations for percent decrease in saturation. **(B)** Comparison of some simulations for percent decrease in concentration. **(C)** Comparison of some simulations for percent decrease in oxygen mass transfer. **(D)** Percent of normal saturation for simulations with variable single apnea duration and constant AHI. **(E)** Percent of normal concentration for simulations with variable single apnea duration and constant AHI.

The decrease for oxygenation in the systemic vessels was compared for some of the simulated cases to assess the relative importance of the AHI and individual apnea duration in determining the severity of decrease in oxygen levels during OSA (Figures 7A–C). For the cases with a constant AHI, the model predicted a positive linear correlation between the apnea duration and the magnitude of percentage decreases (Figures 7D, E). However, there appeared to be no clear correlation between the AHI and percentage decreases (Figures 7A–C).

### 3.7 Performance of model in OSA patients

**FIGURE 8:**
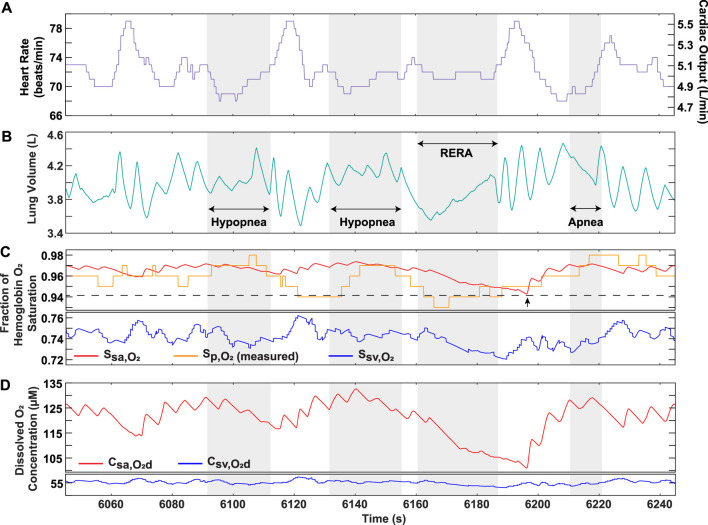
OSA Patient 1 analysis over portion of sleep study. **(A)** Recorded heart rate and cardiac output. **(B)** Lung volume obtained from conversion of recorded nasal pressure. **(C)** Model output hemoglobin oxygen saturation in systemic arteries and veins, along with recorded pulse oximeter data $\left(S_{p,O_{2}}\right)$ . The dashed line and arrow are used to indicate a point where the model predicts a lower value than $S_{p,O_{2}}$ following the RERA event. **(D)** Model output dissolved oxygen concentration in systemic arteries and veins. The grayed portions indicate respiratory events, as labeled in **(B)**. Breaks in the *y*-axis are shown for **(C,D)**.

**FIGURE 9:**
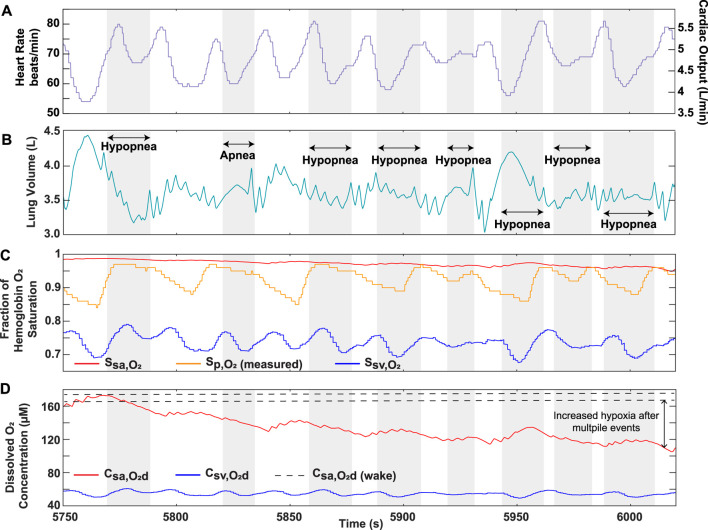
OSA Patient 2 analysis over portion of sleep study. **(A)** Recorded heart rate and cardiac output. **(B)** Lung volume obtained from conversion of recorded nasal pressure. **(C)** Model output hemoglobin oxygen saturation in systemic arteries and veins, along with recorded pulse oximeter data $\left(S_{p,O_{2}}\right)$ . **(D)** Model output dissolved oxygen concentration in systemic arteries and veins. The dashed lines in **(D)** are were used to represent the range of dissolved arterial oxygen during wakefulness. The grayed portions indicate respiratory events, as labeled in **(B)**.

To demonstrate the clinical utility of the model, recorded heart rate and approximated lung volume data from the converted nasal pressure of two OSA patients were used as time-dependent inputs (Supplementary Figures S11, S13). The model output hemoglobin oxygen saturation in the systemic vessels, which can be compared to the recorded pulse oximeter data (Supplementary Figures S12A, S14A). In some regions for Patients 1 and 2, the recorded $S_{p,O_{2}}$ was lower than the arterial saturation predicted by the model, while it was comparatively higher following a respiratory effort related arousal (RERA) event (Figures 8C, 9C), and, in other regions, its fluctuations appeared to be in phase with those of the venous saturation (Figure 9C). In addition, the solution provided the dissolved oxygen concentration in the systemic vessels to better quantify the hypoxic burden on tissues (Supplementary Figures S12B, S14B; Table 4). This gives valuable insight into how the dissolved oxygen in the systemic arteries progressively decreases with continuously occurring obstructive events (Figure 9D). Furthermore, although Patient 2 had a higher AHI score identified in the clinic (calculated as the number of events divided by the total time asleep in hours), the hypoxic burden of Patient 1 was determined to be greater based on the proposed scores (Table 4). This result can be explained by the longer average hypopnea length for Patient 1 from sleep clinic data (Table 4). In addition, the calculated scores allowed for quantification of the overall burden specifically during hypoxic periods in patients, which is valuable insight not reflected in the AHI. For further interpretation of model results, the quantitative analysis performed on data from Patient 1 showed a statistical significance when assessing the differences between the overall and event interval averages for oxygenation (Table 5).

**TABLE 4:** Comparison of model and clinical assessment.

Patient	Proposed burden scores (*μ*M)			
	Event sequence	Total sequence		
	Normalized deviation from wake sequence	Normalized deviation from wake sequence	Normalized deviation from threshold during hypoxia	
1	32	14	18	
2	35	3.3	8.7	

Patient (AHI)	Data from sleep clinic for total sequence			
	Total number of events (apnea/hypopnea)	Total time of events (s)	Average apnea length (s)	Average hypopnea length (s)
1 (11)	17 (3/14)	367	11.4	23.8
2 (53)	34 (5/29)	591	12.5	18.2

**TABLE 5:** Intra-patient statistical analysis.

Analyzed variable	Difference from overall average	*p*-value
Hemoglobin *O*_2_ saturation in systemic arteries	−1.3% ± 0.68%	0.033
Hemoglobin *O*_2_ saturation in systemic veins	−2.2% ± 0.37%	0.001
Difference in dissolved *O*_2_ concentration across systemic capillaries	−18 *μ*M ± 7.4 *μ*M	0.017

Differences reported as mean ± sd. Sample size (*n*) = 4.

## 4 Discussion

The development of cardiovascular consequences in OSA patients is believed to be associated with intermittent hypoxia (Shah et al., 2021). Indeed, changes in tissue oxygenation due to airway obstruction affect the level of cellular reactive oxygen species (ROS), which may lead to vascular injury and remodeling (Shah et al., 2021). Clinical studies have found that the extent of exposure to hypoxia, referred to as “hypoxia burden” in OSA is more predictive of cardiovascular disease than the AHI (Azarbarzin et al., 2019). Considering this, an accurate assessment of OSA severity and the resulting tissue hypoxia requires an understanding of the dissolved oxygen concentration in the blood, which controls the driving force for mass transfer into the tissues. In using the overall metabolism to relate the dissolved oxygen entering and exiting the body tissues, we created a practical and clinically deployable approach to assess tissue oxygenation for different simulations of OSA (Figures 3–6). The model codes are available on GitHub so that anyone can vary the inputs of these simulations for further analysis.

This model was closer in approximating the trend of clinical data when compared to previous literature (Netzer et al., 2001; Cheng and Khoo, 2012). For example, in a previous paper, systemic arterial oxygenation was simulated in response to a generic OSA breathing pattern consisting of four apneas separated by brief periods of hyperventilation (Cheng and Khoo, 2012). A similar breathing pattern with corresponding pulse oximeter data from an OSA patient was presented in another paper (Netzer et al., 2001) and reproduced in our model (Figure 2A). Based on the recorded pulse oximeter data, following each apnea, the minimum arterial hemoglobin saturation drops to a lower value than the one preceding it (Netzer et al., 2001). The trend is also observed in the output of our model (Figure 2), but not in the previous modeling study (Cheng and Khoo, 2012). This is likely due to their assumption of complete equilibration between the alveolar and end-pulmonary capillary oxygen partial pressures (Cheng and Khoo, 2012), which should not be equivalent for such a breathing scenario (Figure 2D). Indeed, this assumption may not be realistic for OSA patients with lower lung oxygen diffusing capacities and for cases of a high heart rate, where reduced red blood cell residence time in the pulmonary capillaries may not allow complete equilibration of partial pressures.

As with any model, there are limitations in our approach, as can be seen in the difference between the predicted systemic arterial saturation $\left(S_{sa,O_{2}}\right)$ and the OSA patient pulse oximetry data (Supplementary Figures S12A, S14A). This is likely caused by the pulse oximetry data itself through inaccuracies in recording due to possible vasoconstriction at the point of measurement or other pulse oximetry errors (Mardirossian and Schneider, 1992). However, it could also indicate the need for an improved estimation of model inputs. For example, our estimation of patient lung volume relied solely on the recorded nasal pressure signal, and the calculation of the fitting parameter used an ideal simulated normal breathing pattern for each patient. As both the tidal volume and FRC may vary with the BMI and positioning of the patient, a more accurate conversion to nasal flow could be done by using proportionality coefficients for inspiration and expiration, determined by recording nasal flow and pressure over a few breaths for each patient (Thurnheer et al., 2001). Another example is that pressure swings may occur during obstructed breathing, which can lead to an inaccurate representation of lung volume (Supplementary Appendix S4; Supplementary Figure S8). In such a scenario, another measure of lung volume, such as chest and abdomen signals from respiratory inductance plethysmography (RIP) during a sleep study, may be used. Additionally, patient-specific approximations of the respiratory anatomy may be useful in determining a more accurate representation of alveolar volume from the lung volume.

The difference between recorded and simulated systemic arterial saturation limitations may also indicate some limitations in our modeling approach. For example, we assume all oxygen transfer in the systemic circulation to occur within the capillaries; however, arteriolar oxygen loss has been observed in previous animal studies (Tsai et al., 2003). This effect is likely to vary among patients, so either more clinical data or an approximation would be needed for model incorporation. Furthermore, in patients with severe obesity, pulmonary shunting resulting from alveolar collapse near the base of the lung (Koenig, 2001) may need to be considered. This can be incorporated into our model by multiplying the pulmonary flow rate by an estimated lung shunt fraction, which would require additional clinical data from a simple chest X-ray.

Currently, the AHI is widely used in clinical practice as an indicator of OSA severity but has been identified as having several limitations (Wu et al., 2016; Osman et al., 2018; Soori et al., 2022), and other parameters have been found to better correlate with disease development and the onset of comorbidities (Wu et al., 2016). Our results elucidate that cases with the same AHI could have vastly different clinical progressions because the tissues are exposed to varying degrees of hypoxia, based on aspects of the breathing pattern not reflected in the AHI (Figure 6). Further, the simulated cases support the proposal to use the duration of obstructive events to assess OSA (Wu et al., 2016), but outputting the blood oxygenation profiles, made available by our model, might capture a fuller clinical picture. For example, there are obstructive events that do not meet prespecified duration and oxygen desaturation criteria mandated by insurance companies, but our model demonstrates that such patients could have a higher hypoxia burden than those with a classical presentation of OSA (Figures 4, 5). These results support the inability of the AHI to accurately capture the physiological changes occurring in the body and indicate that its predetermined criteria may be detrimental to the development of patient treatment plans. Considering this, our model provides an avenue to re-evaluate polysomnography data by using it as an input to predict blood oxygenation for assessment of OSA severity.

For further analysis of OSA, ventilatory response after an apnea was determined to be a factor affecting the blood oxygen concentration (Figure 3). Hyperventilation is commonly observed in OSA patients following an obstructive event due to the onset of hypercapnia (Khoo et al., 1991). Although our model does not currently consider the effects of carbon dioxide, it does demonstrate that hyperventilation following airway obstruction can lead to higher than normal oxygen levels, which is a normal physiological phenomenon called ventilatory overshoot (Figures 3C, D). In addition, the absence of sufficient hyperventilation can result in sustained lower oxygen levels (Figures 3C, D). Both conditions can potentially lead to the onset of pathophysiological processes. Continuously high oxygen exposure of the tissues can cause a state of hyperoxia, which may be detrimental to cellular homeostasis due to the higher production of ROS (Mach et al., 2011). On the other hand, sustained lower oxygen levels can cause tissue hypoxia, which is associated with low ROS levels (Shah et al., 2021). In addition, ventilatory drive is dependent on a patient’s adaptation to hypercapnia, which may differ based on OSA severity (Muraja-Murro et al., 2012). Considering these points, the utility of having clinical respiratory data as an input is essential due to the variation in response to a ventilatory disturbance amongst OSA patients. Although simulated cases of OSA are useful, patient-specific inputs allow for a more accurate assessment of health risks. Our model has this flexibility and is, therefore, proposed as a clinical tool for predicting the effects of OSA. For example, OSA patient gene expression relating to the function of endothelial nitric oxide synthase (eNOS) (Gavrilin et al., 2022), which is affected by ROS levels, could be compared to the duration of hyperoxemia/hypoxemia predicted by our model. This would then allow our model to be used as a tool to estimate gene expression related to hyperoxemia/hypoxemia, while avoiding the need for *in-vitro* polymerase chain reaction (PCR). Such an approximation would give valuable insight into the risk of patients developing pulmonary hypertension and cardiovascular disease.

To further strengthen the applicability of our model as a clinical tool, we demonstrated its ability to predict oxygen levels using OSA patient data as an input. The higher sensitivity of our model solution results from its ability to capture the continuous physiological changes occurring in the body during each breathing cycle, which is not necessarily reflected in the recorded pulse oximeter data (Figure 8C). Furthermore, the model solution displays systemic venous oxygen levels (Supplementary Figures S12, S14), which normally require invasive catheter insertion for direct measurement. Although a patient-specific estimation of the metabolic rate would be needed for accurate prediction, our model provides the utility of venous oxygenation as a potential clinical indicator for assessment of disease severity. In addition, considering the role of dissolved oxygen in controlling the mass transfer gradient to the tissues, the ability of the model to provide systemic arterial and venous dissolved oxygen is important in assessing hypoxia burden. Continuously occurring events lower the driving force for oxygen transfer, a result that cannot be effectively captured by relying on $S_{p,O_{2}}$ (Figures 9C, D). Furthermore, although Patient 2 experiences a greater overall time of obstructive events (Table 4), the lower total hypoxia burden score could indicate that the decrease in airflow during hypopneas is not as severe when compared to Patient 1. This provides a valuable assessment not reflected in the AHI, which does not differentiate hypopnea events based on the severity of airflow restriction. Moreover, the results of the statistical analysis and the patient event series (Figures 8, 9; Table 4) indicate the importance of considering the temporal proximity of obstructive events, another factor not distinctly accounted for in the AHI. The statistical analysis also demonstrates the utility of our model in quantitatively processing data. Our approach can now be used to study large sets of patient data for a more in-depth statistical and clinical analysis.

In this work, we developed a clinically deployable mathematical model to assess OSA. Using various simulated breathing patterns, the results support previous claims of the AHI not being the most reliable predictor of OSA severity. In addition, the clinical application of our model was highlighted by using OSA patient data from multi-hour sleep studies, underscoring several model strengths. With several future directions, we aim to use this model as a tool for evaluating OSA patient health risks. As an example, additional clinically feasible inputs can be incorporated to further improve the accuracy of our model outputs in predicting hypoxic burden on the tissues. Furthermore, an extensive polysomnography data set coupled with our model could allow for a more realistic link between potential clinical indicators of OSA and disease progression. The results could then be compared to gene expression in patients for further analysis. In addition, narrowing our analysis on certain target organs could allow the model to identify the most likely comorbidity presentation in OSA patients. With these future avenues, we will aim to use our model for the improvement of patient care.

## Data Availability

The original contributions presented in the study are included in the article/Supplementary Material, further inquiries can be directed to the corresponding authors.
