# Supplementary material for: A mathematical model to serve as a clinical tool for assessing obstructive sleep apnea severity
Source: Front Physiol. 2023 Aug 3;14:1198132. doi: 10.3389/fphys.2023.1198132 (PMC10434550; doi:10.3389/fphys.2023.1198132)
Supplement: Supplementary file 1 [file DataSheet1.pdf]

frontiers

Supplementary Material

CONTENTS

1 Derivations and Finite Difference Forms of Equations 2
- 1.1 Pulmonary Arterial Oxygenation 9
- 1.2 Pulmonary Capillary Mass Transfer 11
- 1.3 Alveolar Oxygenation 13

2 Initial Condition for Pulmonary Capillary Profile 13

3 Assumption of Pseudo-Steady State Diffusion Across Alveolar-Capillary Membrane 14

4 Estimation of OSA Patient Lung Volume Using Recorded Nasal Pressure 14

5 MATLAB Implementation 18
- 5.1 Pulmonary Arterial Oxygenation 18
- 5.2 Pulmonary Capillary Mass Transfer 19
- 5.3 Alveolar Mass Transfer 20
- 5.4 Time Step for Simulations and Patient Cases 20
- 5.5 Estimation of OSA Patient Lung Volume Using Recorded Nasal Pressure 20
- 5.6 Lookup Tables 21

6 Model Outputs 22
- 6.1 Comparison of Normal Breathing Results with Expected Physiological Values 22
- 6.2 Calculation of Percent Decreases for OSA Simulations 22
- 6.3 Calculation of Proposed Burden Scores 23
- 6.4 Extended Data Set for OSA Patient Sleep Studies 25
- 6.5 Statistical Analysis 29

1 DERIVATIONS AND FINITE DIFFERENCE FORMS OF EQUATIONS

Table S1: Model Variables and Terms (in Main Paper)

Variable	Definition	Equation	Units in Model	Initial Condition (Source)
C_{A,O_2}	Dissolved oxygen concentration in alveolar membrane	Eq. 4c	mol/mL	Eq. 4c
C_{c,O_2}	Spatially averaged dissolved oxygen concentration in pulmonary capillary compartment	Eq. 5c	mol/mL	Eq. 5c
$C_{cv,d}$	Dissolved oxygen concentration in control volume	Eq. 4	mol/mL	N/A
$C_{cv,T}$	Total oxygen concentration in control volume	Eq. S2 (Systemic capillary compartment) or S7 (Pulmonary capillary compartment)	mol/mL	N/A
C_{pa,O_2d}	Dissolved oxygen concentration in pulmonary arteries	Eq. 2 solved for C_{pa,O_2d}	mol/mL	Eq. 2 solved for C_{pa,O_2d}
C_{pa,O_2T}	Total oxygen concentration in pulmonary arteries	Eq. S4	mol/mL	Eq. S4
C_{pc,O_2d}	Dissolved oxygen concentration in pulmonary capillary compartment	Eq. 2 solved for C_{pc,O_2d}	mol/mL	Eq. 2 solved for C_{pc,O_2d}
C_{pc,O_2T}	Total oxygen concentration in pulmonary capillary compartment	Eq. S8	mol/mL	Eq. S14
C_{pv,O_2d}	Dissolved oxygen concentration in pulmonary veins	Eq. 2 solved for C_{pv,O_2d}	mol/mL	Eq. 3a solved for C_{pv,O_2d}
C_{pv,O_2T}	Total oxygen concentration in pulmonary veins	Eq. S11	mol/mL	Eq. 2
C_{sa,O_2d}	Dissolved oxygen concentration in systemic arteries	Eq. 2 solved for C_{sa,O_2d}	mol/mL	Eq. 3a solved for C_{sa,O_2d}
C_{sa,O_2T}	Total oxygen concentration in systemic arteries	Eq. S5	mol/mL	Eq. 2

Table S1: continued

Variable	Definition	Equation	Units in Model	Initial Condition (Source)
C_{sv,O_2d}	Dissolved oxygen concentration in systemic veins	Eq. 2 solved for C_{sv,O_2d}	mol/mL	Eq. 2 solved for C_{sv,O_2d}
C_{sv,O_2T}	Total oxygen concentration in systemic veins	Eq. S3	mol/mL	Eq. S3
P_{A,O_2}	Alveolar oxygen partial pressure	Eqs. S12 (Inspiration) and S13 (Expiration)	mmHg	104 mmHg (Guyton and Hall (2000))
P_{pa,O_2}	Oxygen partial pressure in pulmonary arteries	Eq. 3a	mmHg	Eq. 3a
P_{pc,O_2}	Oxygen partial pressure in pulmonary capillary compartment	Eq. 3a	mmHg	Eq. 3a
P_{pv,O_2}	Oxygen partial pressure in pulmonary veins	Eq. 3a	mmHg	104 mmHg (Guyton and Hall (2000))
P_{sa,O_2}	Oxygen partial pressure in systemic arteries	Eq. 3a	mmHg	104 mmHg (Guyton and Hall (2000))
P_{sv,O_2}	Oxygen partial pressure in systemic veins	Eq. 3a	mmHg	Eq. 3a
S_{pa,O_2}	Fraction of hemoglobin saturation in pulmonary arteries	Eq. 3	Unitless	Eq. 3
S_{pc,O_2}	Fraction of hemoglobin oxygen saturation in pulmonary capillary compartment	Eq. 3	Unitless	Eq. 3
S_{pv,O_2}	Fraction of hemoglobin oxygen saturation in pulmonary veins	Eq. 3	Unitless	Eq. 3
S_{sa,O_2}	Fraction of hemoglobin oxygen saturation in systemic arteries	Eq. 3	Unitless	Eq. 3
S_{sv,O_2}	Fraction of hemoglobin oxygen saturation in systemic veins	Eq. 3	Unitless	Eq. 3
t	Time	Independent variable	s	0 s

Table S1: continued

Variable	Definition	Equation	Units in Model	Initial Condition (Source)
ΔV	Differential volume	Eq. S1a (Systemic capillary compartment) or S6a (Pulmonary capillary compartment)	mL	N/A
V_A	Simulated alveolar volume	Normal, Severe OSA, Sim. 1-9: Variation of Eq. 7, Patient Cases: Eq. S17	mL	Normal, Severe OSA, Sim. 1-9: 2,300 mL (Reynolds et al. (2010)), Patient Case 1: 3,700 mL and Patient Case 2: 3,400 mL (Abdeyrim et al. (2015))
z	Spatial coordinate	Independent variable	cm	N/A

Table S2: Physiological Parameters (in Main Paper)

Parameter	Definition	Value (Source)/Equation	Units in Model
A_{eff}	Effective cross-sectional area of all pulmonary capillaries	3,000 cm^2 (Aird (2005); Boron and Boulpaep (2009); Guyton and Hall (2000))	cm^2
β_p	Oxygen solubility in alveolar-capillary membrane and blood plasma	1.4×10^{-9} mol $O_2 \cdot$ mL$^{-1} \cdot$ mmHg^{-1} (Frank et al. (1997))	mol $O_2 \cdot$ mL$^{-1} \cdot$ mmHg^{-1}
b_r	Breathing rate	Normal, Severe OSA, Sim. 1-9, and Sim. Patient Case 2: 12 breaths/min, Sim. Patient Case 1: 20 breaths/min, Severe OSA: 24 breaths/min (hyperventilation), Sim. 2: 24 breaths/min (hyperventilation)	breaths/s

Table S2: continued

Parameter	Definition	Value (Source)/Equation	Units in Model
C_{Hb}	Hemoglobin concentration in blood	2.3×10^{-6} mol/mL (Dash and Bassingthwaighte (2010))	mol/mL
D_{L,O_2}	Normal oxygen lung diffusing capacity	21 mL $O_2 \cdot \text{min}^{-1} \cdot \text{mmHg}^{-1}$ (Guyton and Hall (2000))	mol $O_2 \cdot \text{s}^{-1} \cdot \text{mmHg}^{-1}$
k_l	Lung mass transfer coefficient	Eq. 5b	cm^3/s
k_{pc}	Pulmonary capillary control volume mass transfer coefficient	Eq. 4a	cm^3/s
L_c	Pulmonary capillary compartment length	Eq. 4b	cm
MR_{O_2}	Total basal metabolic rate for oxygen consumption	240 mL O_2/min (MacIntyre (2014))	mol O_2/s
P_B	Barometric pressure	760 mmHg (Guyton and Hall (2000))	mmHg
P_{H_2O}	Water vapor pressure at normal body temperature	47 mmHg (Guyton and Hall (2000))	mmHg
P_{I,O_2}	Inspired oxygen partial pressure	Eq. 5a	mmHg
R	Ideal gas constant	62,360 $\text{mL} \cdot \text{mmHg} \cdot \text{mol}^{-1} \cdot \text{K}^{-1}$	$\text{mL} \cdot \text{mmHg} \cdot \text{mol}^{-1} \cdot \text{K}^{-1}$
T	Normal body temperature	37°C	K
V_D	Dead space volume	150 mL (Reynolds et al. (2010))	mL
V_{End}	End-expiration alveolar volume	Normal, Severe OSA, Sim. 1-9: 2,300 mL (Reynolds et al. (2010)), Patient Case 1: 3,700 mL and Patient Case 2: 3,400 mL (Abdeyrim et al. (2015))	mL
V_{pc}	Total pulmonary capillary blood volume	130 mL (Ben-Tal (2006); Foucquier et al. (2013))	mL
$V_{sys,cap}$	Total systemic capillaries blood volume	330 mL (Boron and Boulpaep (2009))	mL

Table S2: continued

Parameter	Definition	Value (Source)/Equation	Units in Model
V_T	Tidal volume	Normal and Sim. 1-9: 500 mL (Reynolds et al. (2010)), Severe OSA: 1,000 mL (Cheng and Khoo (2012)), Sim. Patient Case 1: 510 mL and Sim. Patient Case 2: 560 mL (ARD (2014))	mL
V_{vent}	Alveolar ventilation volume	Eq. 7a	mL
y_{O_2}	Inspired oxygen mole fraction	0.21 (Guyton and Hall (2000))	Unitless

Table S3: Additional Model Variables and Terms

Variable/Term	Description	Equation	Units in Model	Initial Condition (Source)
C_{d,O_2}	Dissolved oxygen concentration	Eq. S15	mol/mL	N/A
$J_{O_2,x}$	Molar flux of oxygen across alveolar-capillary membrane	Eq. S9	mol/cm^2	Eq. S9
HR	Heart rate	Normal, Severe OSA, Sim. 1-9: Constant, Patient Cases: Clinical data	beats/s	73 beats/min (Cheng and Khoo (2012))
P_{c,O_2}	Average oxygen partial pressure in pulmonary capillary compartment	Eq. S12a	mmHg	Eq. S12a
P_N	Normalized OSA patient nasal pressure	Eq. S31	cm H_2O	Input
$P_{N,m}$	Measured OSA patient nasal pressure	Clinical data	cm H_2O	Input
Q_N	OSA patient nasal flow	Eq. S20	mL/s	Input

Q_p	Pulmonary flow rate	Normal, Severe OSA, Sim. 1-9: Constant, Patient Cases: Eq. S6b	mL/s	Eq. S6b
Q_s	Systemic flow rate	Normal, Severe OSA, Sim. 1-9: Constant, Patient Cases: Eq. S1b	mL/s	Eq. S1b
Δt	Differential time step	Normal, Severe OSA, Sim. 1-9: Constant, Patient Cases: Eq. S1c	s	Normal, Severe OSA, Sim. 1-9: 0.01 s, Patient Case 1: 0.008 s, Patient Case 2: 0.007 s
$V_{A,OSA}$	OSA patient alveolar volume	Eq. S21	mL	Case 1: 3,700 mL and Case 2: 3,400 mL (Abdeyrim et al. (2015))
v_{pc}	Pulmonary capillary blood velocity	Normal, Severe OSA, Sim. 1-9: Constant, Patient Cases: Eq. S8b	cm/s	Eq. S8b

Table S4: Additional Physiological and Fitting Parameters

**Parameter**	**Definition**	**Value (Source)/Equation**	**Units in Model**
A	Total surface area for gas exchange in lungs, at rest	84 m^2 (Hsia et al. (2016))	cm^2
D_{eff}	Effective oxygen diffusion coefficient	Eq. S9a	cm^2/s
γ	Fitting parameter for conversion of patient nasal pressure to nasal flow	Eq. S19	mL $\cdot$ s^{-1} $\cdot$ cm H_2O^{-1}
l_{mem}	Harmonic mean thickness of alveolar-capillary membrane	0.6 μm (Hsia et al. (2016))	cm
n_V	Molar volume at STP	1/22,400 mol O_2/mL O_2	mol O_2/mL O_2
SV	Stroke volume	70 mL/beat (Bruss and Raja (2022))	mL/beat

Table S4: continued

Parameter	Definition	Value (Source)/Equation	Units in Model
t_{cycle}	Total time for control volume of blood to pass through systemic circulation	Eq. S1d	s
t_d	Diffusion time across alveolar-capillary membrane	Eq. S16	s
t_{sa}	Total time for control volume of blood to pass through systemic arteries/arterioles	Eq. S5a	s
$t_{sys,cap}$	Total time for control volume of blood to pass through systemic capillaries	Eq. S3a	s
V_{sys}	Systemic circulation blood volume	4,600 mL (Boron and Boulpaep (2009))	mL
$V_{sys,a}$	Systemic arteries/arterioles blood volume	770 mL (Boron and Boulpaep (2009))	mL
Δz	Differential distance	Eq. S8a	cm

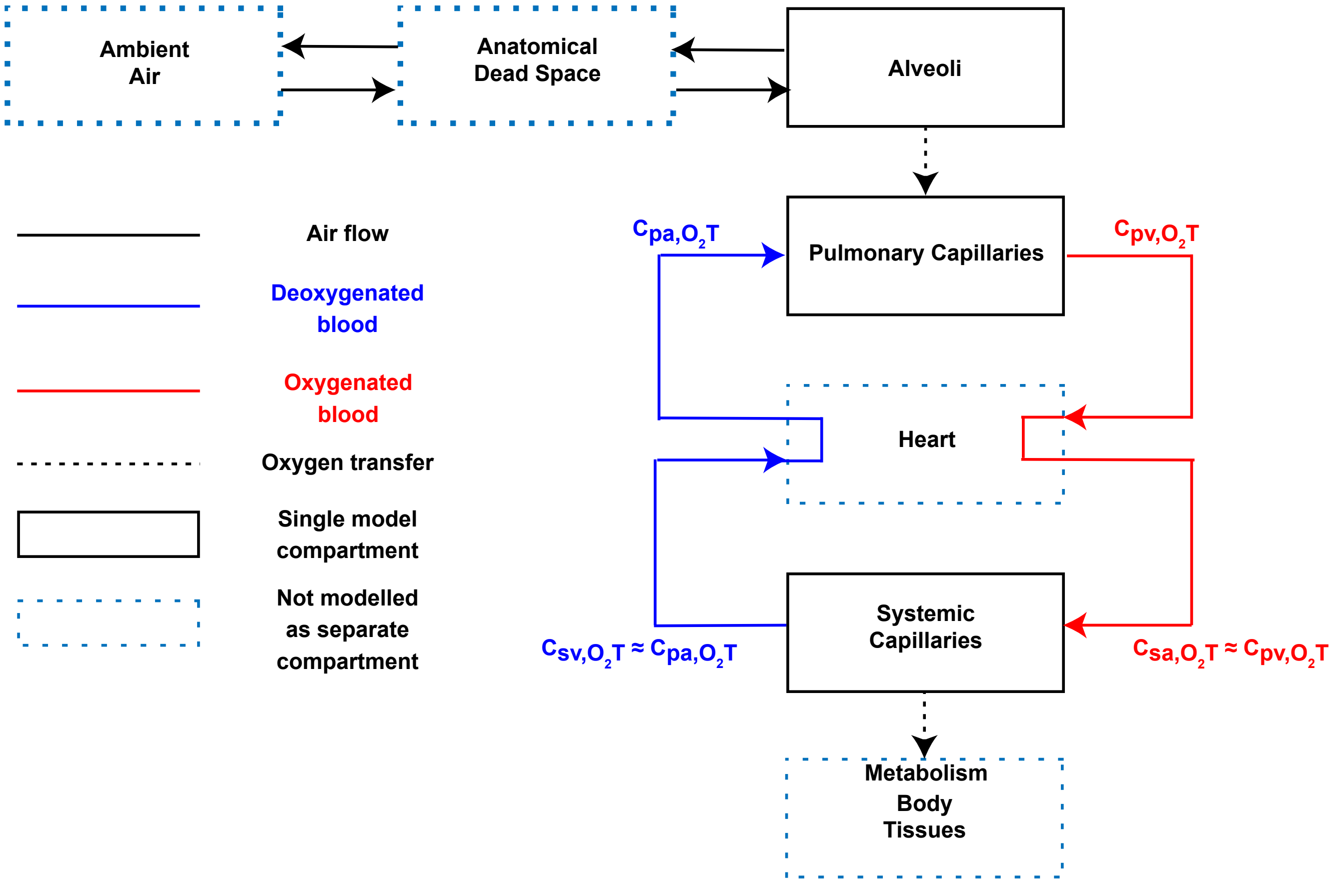

Figure S1. A flow diagram depicting the model physiology. The alveoli, pulmonary capillaries, and systemic capillaries were modeled as single compartments. Parameters related to the ambient air (external environment), anatomical dead space, heart, and body tissues were included in the model, but no mass balances were performed over these regions. All oxygen transfer in the systemic circulation was assumed to occur in the capillary region. Therefore, the pulmonary venous and arterial total oxygen concentrations were assumed to be equivalent to the systemic arterial and venous total oxygen concentrations, respectively.

1.1 Pulmonary Arterial Oxygenation

Equation 1, as restated below in its expanded form, was obtained by performing an unsteady-state mass balance over a moving control volume in the systemic capillary compartment, with an overall metabolic rate used for modeling oxygen transfer to the body tissues. To determine the mass transfer only occurring out of the control volume, the overall oxygen metabolic rate was multiplied by a ratio quantifying the fraction of the systemic capillary compartment occupied by the control volume:

$$\Delta V \frac{dC_{cv,T}}{dt} = -MR_{O_2} \left(\frac{\Delta V}{V_{sys,cap}} \right)$$

$$\Delta V = Q_s(t) \cdot \Delta t(t) \qquad \text{(S1a)} \qquad\qquad Q_s(t) = HR(t) \cdot SV \qquad \text{(S1b)}$$

$$\Delta t(t) = \frac{t_{cycle}(t) \cdot \Delta t(t = 0)}{t_{cycle}(t = 0)} \quad \text{(S1c)} \qquad t_{cycle}(t) = \frac{V_{sys}}{Q_s(t)} \quad \text{(S1d)}$$

For the OSA patient cases, the systemic blood flow is a function of time as it varies with the input heart rate (Equation S1b). In addition, since Δt was made to vary proportionally with the heart rate, for data storage purposes (Appendix S5.1), ΔV remains constant. The finite difference form of Equation 1 is:

$$C_{cv,T}(t + \Delta t) = C_{cv,T}(t) - MR_{O_2}\left(\frac{\Delta t}{V_{sys,cap}}\right) \quad \text{(S2)}$$

Solving Equation S2 over time resulted in the following relation between the total oxygen concentration exiting and entering the systemic capillary compartment:

$$C_{sv,O_2T}(t + t_{sys,cap}(t)) = C_{sa,O_2T}(t) - \frac{MR_{O_2}}{Q_s(t)} \quad \text{(S3)}$$

$$t_{sys,cap}(t) = \frac{V_{sys,cap}}{Q_s(t)} \quad \text{(S3a)}$$

Assuming that all oxygen transfer in the systemic circulation occurs in the capillary region, the total oxygen concentration in the pulmonary arteries and veins was related as:

$$C_{pa,O_2T}(t + t_{cycle}(t)) = C_{pv,O_2T}(t) - \frac{MR_{O_2}}{Q_s(t)} \quad \text{(S4)}$$

In addition, the total oxygen concentration in the pulmonary veins and that entering the systemic capillary compartment were related in time as:

$$C_{sa,O_2T}(t + t_{sa}(t)) = C_{pv,O_2T}(t) \quad \text{(S5)}$$

$$t_{sa}(t) = \frac{V_{sys,a}}{Q_s(t)} \quad \text{(S5a)}$$

This assumes no oxygen leakage in the arteriole system. If there is possible arteriolar oxygen transfer to the tissues, the model will be adjusted accordingly. Due to their dependence on the systemic flow rate, $t_{sys,cap}$, t_{cycle}, and t_{sa} were all functions of time for the patient cases. However, for the simulations, Q_s, Δt, $t_{sys,cap}$, t_{cycle}, and t_{sa} were all constant due to an assumed constant heart rate (Table S3).

1.2 Pulmonary Capillary Mass Transfer

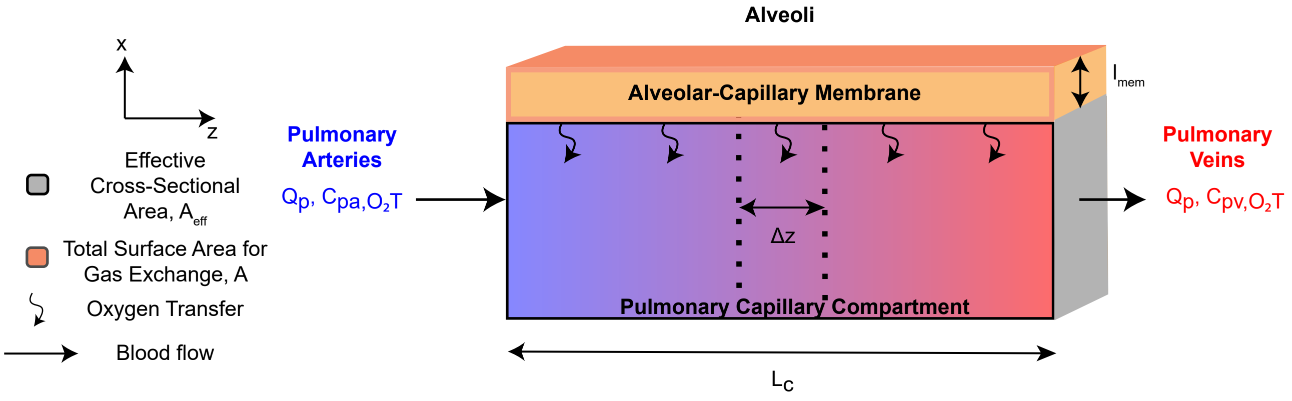

Figure S2. A schematic of the pulmonary capillary compartment. Deoxygenated blood enters from the pulmonary arteries and is oxygenated as a result of diffusion across the alveolar-capillary membrane. Δz is the length of the differential control volume. Since the overall pulmonary flow passes through the compartment, an effective cross-sectional area is used (sum of all pulmonary capillaries), A_{eff}, to determine the compartment length, L_c. Oxygenated blood exits the compartment in the pulmonary veins.

Equation 4, as restated below, was obtained by performing an unsteady-state mass balance over a moving control volume in the pulmonary capillary compartment:

$$\Delta V \frac{dC_{cv,T}}{dt} = k_{pc}\Big(C_{A,O_2}(t) - C_{cv,d}(t)\Big)$$

$$\Delta V = Q_p(t) \cdot \Delta t(t) \qquad \text{(S6a)} \qquad\qquad Q_p(t) = HR(t) \cdot SV \qquad \text{(S6b)}$$

For the OSA patient cases, the pulmonary blood flow is a function of time as it varies with the input heart rate (Equation S6b). Considering that the total pulmonary and systemic flow rates are equal to the cardiac output, ΔV for the pulmonary capillary compartment is constant due to the varying Δt.

The finite difference form of Equation 4 is:

$$C_{cv,T}(t) = C_{cv,T}(t - \Delta t) + \frac{k_{pc}}{Q_p(t - \Delta t)}\Big(C_{A,O_2}(t - \Delta t) - C_{cv,d}(t - \Delta t)\Big) \qquad \text{(S7)}$$

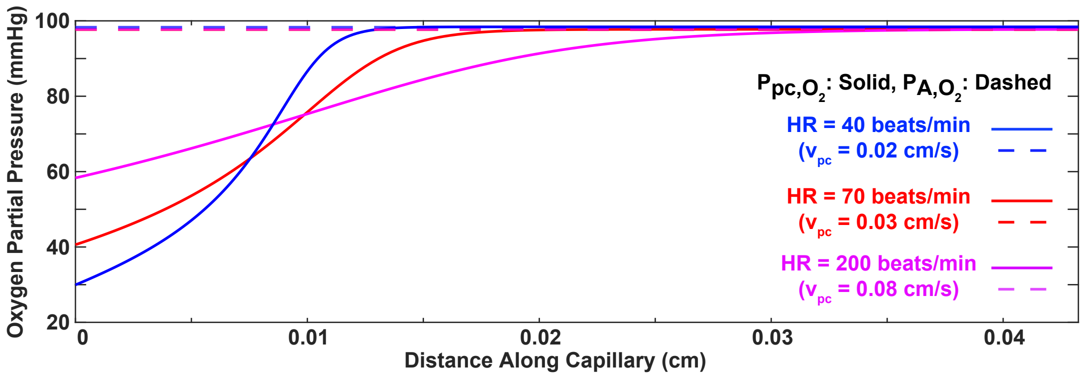

Figure S3. Effect of perfusion on model results. As the heart rate is increased, equilibration between the alveolar (dashed lines) and pulmonary capillary (solid lines) oxygen partial pressures occurs further along the capillary due to reduced residence time, indicating the limiting effect of perfusion in mass transfer.

To obtain an oxygen profile along the pulmonary capillaries, the starting boundary of the control volume was used to define its location. The pulmonary blood velocity was then used to track the movement of this volume along the pulmonary capillary compartment, introducing spatial dimensionality into the model and ensuring the significant influence of perfusion, which controls the equilibration between alveolar and end-capillary oxygen partial pressure (demonstrated with Figure S3).As a result, Equation S7 was transformed into:

$$C_{pc,O_2T}(z,t) = C_{pc,O_2T}(z-\Delta z, t-\Delta t) + \frac{k_{pc}}{Q_p(t-\Delta t)}\Big(C_{A,O_2}(t-\Delta t) - C_{pc,O_2d}(z-\Delta z, t-\Delta t)\Big) \quad \text{(S8)}$$

$$\Delta z = v_{pc}(t)\cdot \Delta t(t) \quad \text{(S8a)} \qquad v_{pc}(t) = \frac{Q_p(t)}{A_{eff}} \quad \text{(S8b)}$$

Due to its dependence on the pulmonary flow rate, v_{pc} is also function of time for the patient cases. However, since Δt varies proportionally with the heart rate, Δz remains constant. Q_p, Δt, and v_{pc} were kept constant for all the simulated cases due to an assumed constant heart rate (Table S3). Oxygen transfer into the control volume was modeled assuming a pseudo-steady state diffusion process across the alveolar-capillary membrane:

$$J_{O_2,x}(z,t) = \frac{D_{eff}}{l_{mem}}\big(C_{A,O_2}(t) - C_{pc,O_2d}(z,t)\big) \quad \text{(S9)}$$

$$D_{eff} = \frac{D_{L,O_2}\cdot l_{mem}}{\beta_p \cdot A} \quad \text{(S9a)}$$

In addition, the total oxygen concentration at the entrance of the pulmonary capillary compartment was set as pulmonary arterial value, while the total oxygen concentration in the pulmonary veins was set as the end-capillary value.

$$C_{pc,O_2T}(0,t) = C_{pa,O_2T}(t) \quad \text{(S10)}$$

$$C_{pv,O_2T}(t) = C_{pc,O_2T}(L_c,t) \quad \text{(S11)}$$

1.3 Alveolar Oxygenation

Converting Equation 5 to a finite difference form enabled efficient calculation of the alveolar oxygen partial pressure at any time point during inspiration. This equation was used under the condition that $\dfrac{V_A(t) - V_A(t-\Delta t)}{\Delta t} > 0$.

$$P_{A,O_2}(t) = \frac{\Big(V_A(t) - V_A(t-\Delta t)\Big)\Big(P_{I,O_2} - P_{A,O_2}(t-\Delta t)\Big)}{V_A(t-\Delta t)} - \frac{k_l \cdot \beta_p \cdot \Delta t\Big(P_{A,O_2}(t-\Delta t) - P_{c,O_2}(t-\Delta t)\Big)R \cdot T}{V_A(t-\Delta t)} + P_{A,O_2}(t-\Delta t) \tag{S12}$$

$$P_{c,O_2}(t) = \frac{C_{c,O_2}(t)}{\beta_p} \tag{S12a}$$

Converting Equation 6 to a finite difference form enabled efficient calculation of the alveolar oxygen partial pressure at any time point during expiration. This equation was used under the condition that $\dfrac{V_A(t) - V_A(t-\Delta t)}{\Delta t} < 0$.

$$P_{A,O_2}(t) = -\frac{k_l \cdot \beta_p \cdot \Delta t\Big(P_{A,O_2}(t-\Delta t) - P_{c,O_2}(t-\Delta t)\Big)R \cdot T}{V_A(t-\Delta t)} + P_{A,O_2}(t-\Delta t) \tag{S13}$$

Δt was kept constant for all the simulations but varied for the OSA patient cases according to Equation S1c.

2 INITIAL CONDITION FOR PULMONARY CAPILLARY PROFILE

As an approximation, the initial oxygen profile (t=0) along the pulmonary capillary compartment was determined by solving a steady state mass balance over a fixed control volume.

$$C_{pc,O_2T}(z) = C_{pc,O_2T}(z-\Delta z) + \frac{k_{pc}}{Q_p(t=0)}\left(C_{A,O_2}(t=0) - C_{pc,O_2d}(z-\Delta z)\right) \tag{S14}$$

The initial conditions for the total and dissolved oxygen concentrations in the pulmonary arteries were used for $C_{pc,O_2T}(z=0)$ and $C_{pc,O_2d}(z=0)$, respectively. The starting value for the total oxygen in the pulmonary arteries was calculated using Equation S4. As an additional form of validation, the predicted average oxygen partial pressure in the pulmonary arteries (P_{pa,O_2} = 41 mmHg) approximates the expected physiological value of 40 mmHg (Guyton and Hall (2000)) for a normal subject at rest.

3 ASSUMPTION OF PSEUDO-STEADY STATE DIFFUSION ACROSS ALVEOLAR-CAPILLARY MEMBRANE

The molar flux of oxygen across the alveolar-capillary membrane can be represented as (Popel (1989)):

$$J_{O_2,x}(z,t) = -D_{\mathit{eff}} \frac{dC_{d,O_2}}{dx} \tag{S15}$$

Equation S9a was used as an approximation of the effective diffusion coefficient, which was applied to estimate the diffusion time across the alveolar-capillary membrane:

$$t_d = \frac{l_{mem}^2}{D_{\mathit{eff}}} = \frac{\left(0.6 \times 10^{-4}\ \mathrm{cm}\right)^2}{8 \times 10^{-7}\ \mathrm{cm}^2/\mathrm{s}} = 0.005\ \mathrm{s} \tag{S16}$$

The process time (time for boundary changes) was taken to be on the order of the time for one breath. With a normal breathing rate of 12 breaths/min, this would be 5 s, while, with a hyperventilatory rate of 24 breaths/min, the process time would be 2.5 s. For both of these cases, the process time was concluded to be sufficiently larger than the diffusion time, allowing for the pseudo-steady state simplification.

4 ESTIMATION OF OSA PATIENT LUNG VOLUME USING RECORDED NASAL PRESSURE

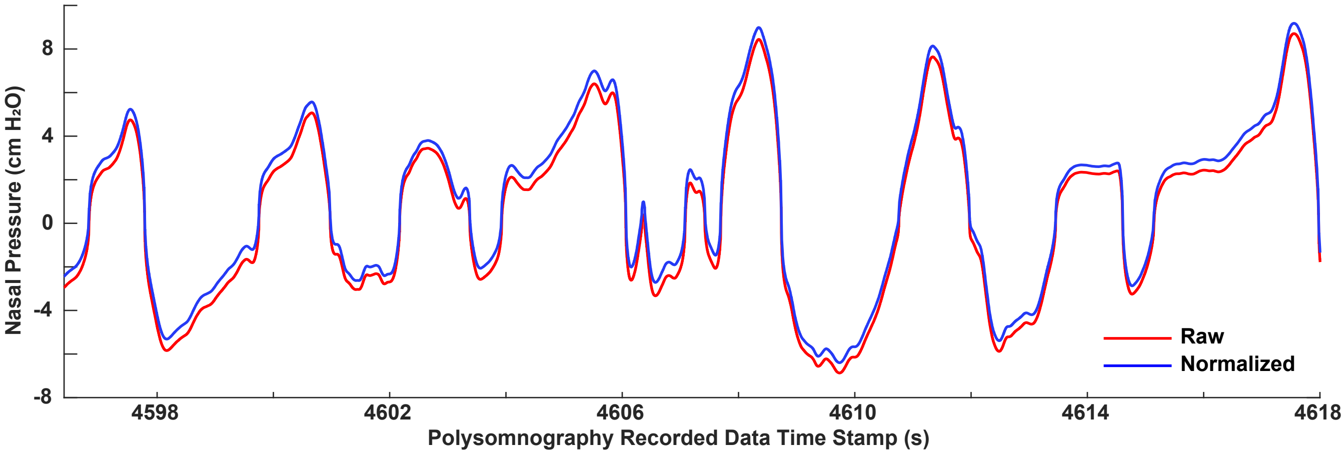

Figure S4. Patient 1 raw and normalized nasal pressure signals over isolated normal breathing region.

For each patient, the recorded pressure signal was observed to have a non-zero mean, which resulted in unphysiological values for the lung volume after conversion. Therefore, to allow for a more realistic representation of the volume, each pressure signal was normalized to a mean of zero by subtracting a moving average taken over 70 seconds for Patient 1 and 50 seconds for Patient 2. The time for each moving average was chosen such that it was on the order of a few breaths, while ensuring that the normalized signal maintained the integrity of the raw pressure signal (Figures S4 and S5).

The following equations were used to simulate normal breathing patterns and corresponding flow rates for the OSA patients, with the tidal volume and breathing rate for each determined using the patient

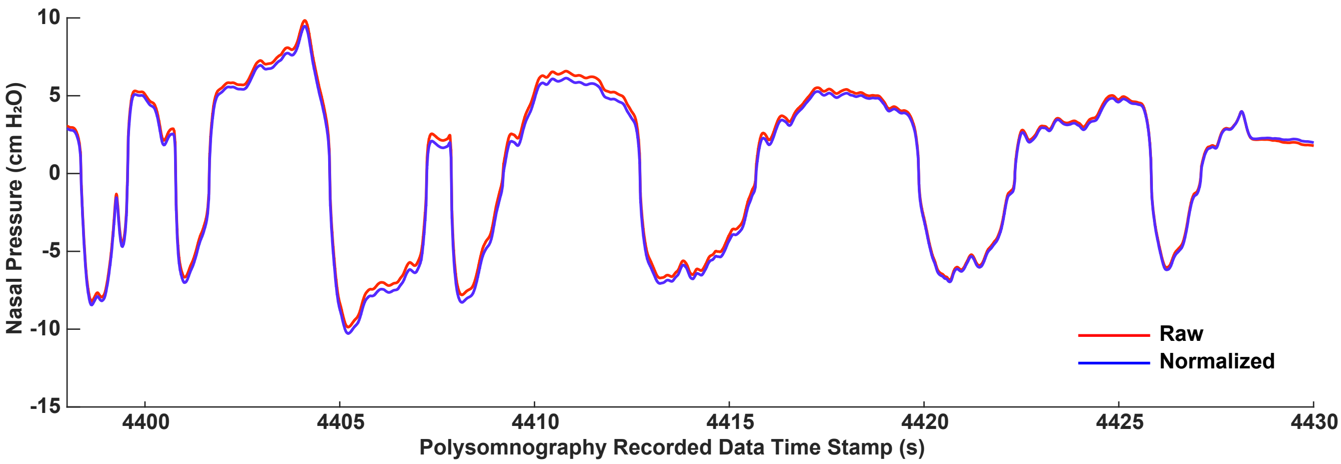

Figure S5. Patient 2 raw and normalized nasal pressure signals over isolated normal breathing region.

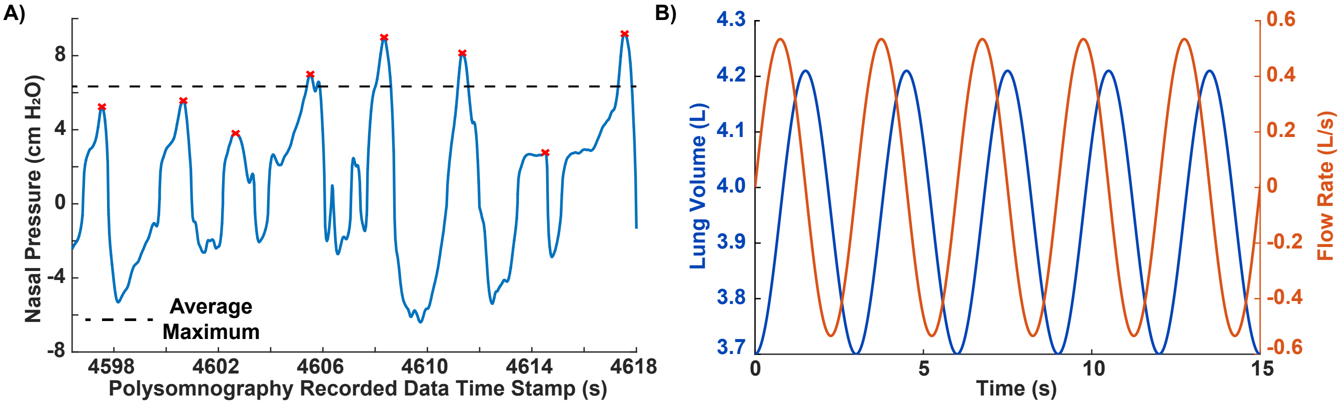

Figure S6. Actual and simulated signals for Patient 1. A) Normalized patient nasal pressure signal during isolated normal breathing sequence. B) Simulated normal lung volume and flow rate using breathing rate from isolated patient signal ($\bar{b}_r$ = 20 breaths/min) and tidal volume from patient height (V_T = 0.51 L).

height-based ideal body weight and normalized pressure signal, respectively:

$$V_A(t) \approx \frac{1}{2} V_T \sin\left(2\pi \cdot b_r \cdot t - \frac{\pi}{2}\right) + \left(\frac{1}{2} V_T + V_{End}\right) \tag{S17}$$

and

$$\frac{dV_A}{dt} \approx \pi \cdot b_r \cdot V_T \cos\left(2\pi \cdot b_r \cdot t - \frac{\pi}{2}\right) \tag{S18}$$

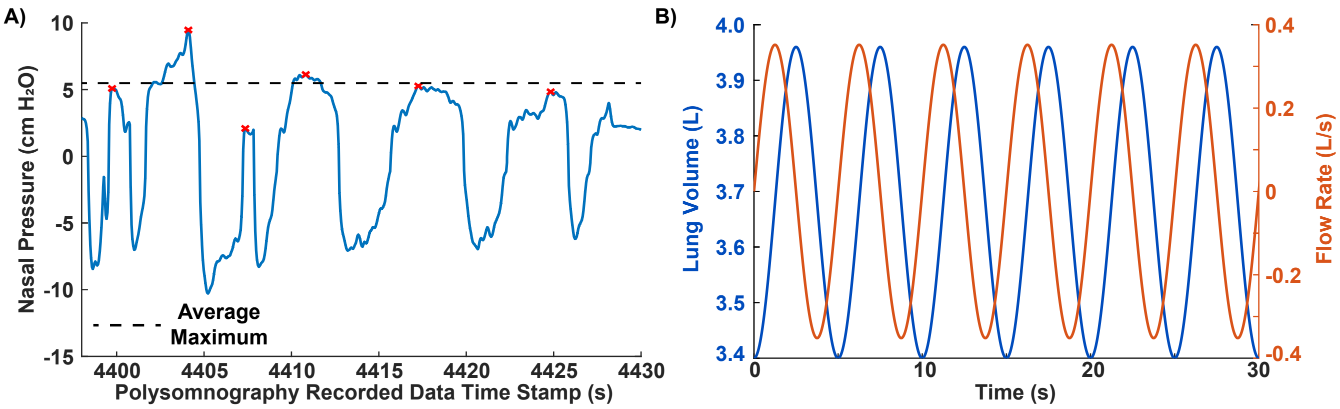

Figure S7. Actual and simulated signals for Patient 2. A) Normalized patient nasal pressure signal during isolated normal breathing sequence. B) Simulated normal lung volume and flow rate using breathing rate from isolated patient signal (b_r = 12 breaths/min) and tidal volume from patient height (V_T = 0.56 L).

A positive nasal pressure indicated inspiration, while a negative nasal pressure indicated expiration. Therefore, for each patient, the fitting parameter for conversion to nasal flow was determined by setting the average maximum nasal pressure (after normalization) during the isolated normal breathing sequence (Figures S6A and S7A) to the maximum simulated inspiratory flow rate (Figures S6B and S7B):

$$\gamma = \frac{\pi \cdot b_r \cdot V_T}{P_{N\{\text{norm,avg.max}\}}} \tag{S19}$$

Each normalized nasal pressure signal was converted to nasal flow as:

$$Q_N(t) = \gamma \cdot P_N(t) \tag{S20}$$

The patient alveolar volume, approximated as the lung volume, was determined by integration of the obtained flow signal:

$$V_{A,OSA}(t) = V_{End} + \int Q_N(t)\,\mathrm{d}t \tag{S21}$$

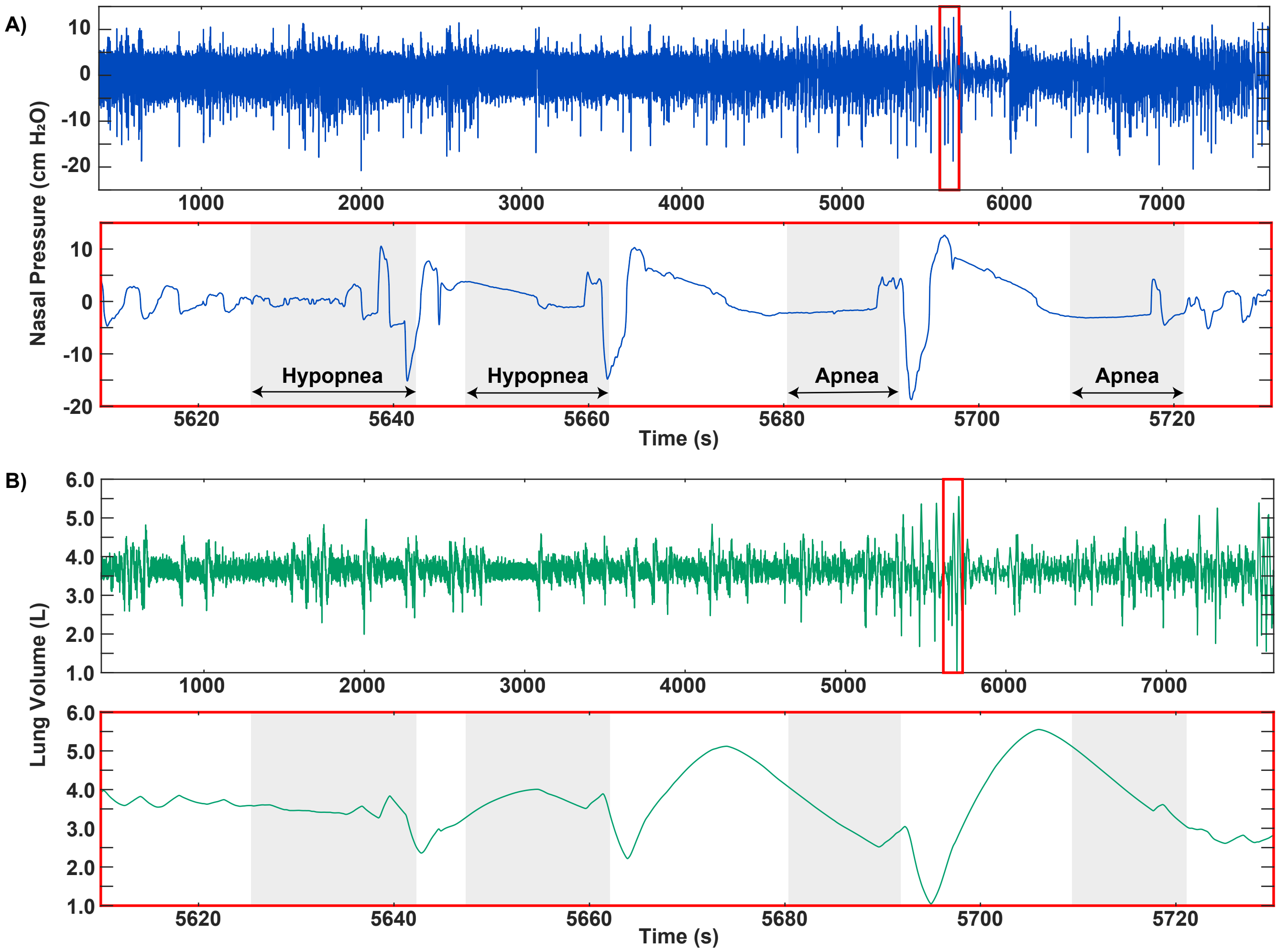

Figure S8. Pressure swings during a segment with obstructive events. A) Raw pressure signal during polysomnography study. B) Simulated lung volume after converting normalized pressure using fitting parameter. The sections outlined in red show a sequence with multiple obstructive events and drastic pressure swings, resulting in a low lung volume after conversion. The grayed portions indicate obstructive respiratory events, as labeled in (A).

While analyzing Patient 2, a region with multiple obstructive events and drastic pressure swings was observed (Figure S8A, outlined in red). Although the majority of the volume signal after conversion is reasonable, this section is an example where relying solely on nasal pressure results in values of the simulated lung volume that are much lower than the observed residual volume in OSA patients (Abdeyrim et al. (2015)), with the lowest point dropping close to 1.0 L (Figure S8B). Using RIP chest and abdomen signals, which are available within most clinical polysomnographies, in conjunction with the nasal pressure will likely provide a more accurate representation of patient lung volume in such cases.

5 MATLAB IMPLEMENTATION

5.1 Pulmonary Arterial Oxygenation

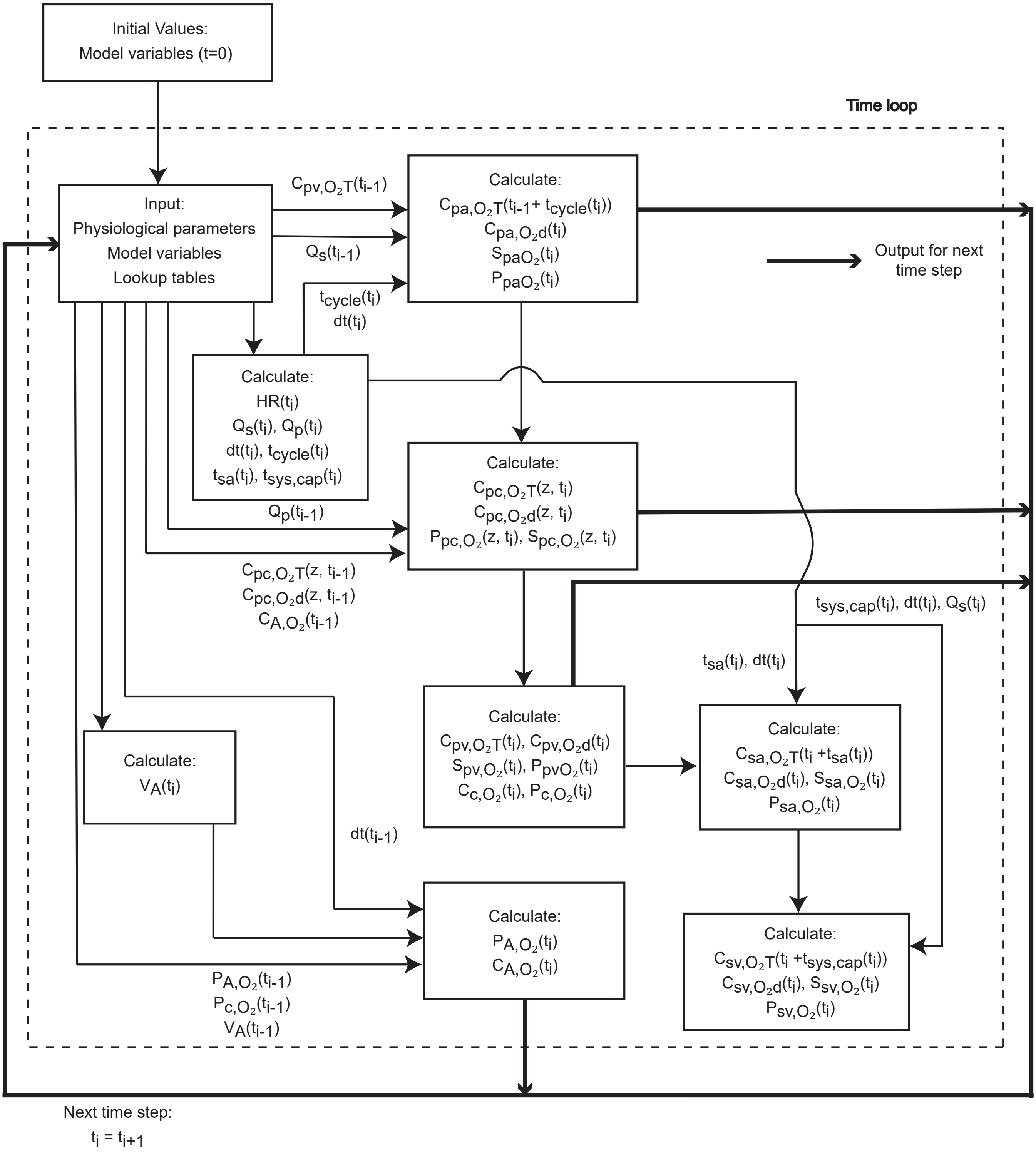

Figure S9. Model framework. Breathing and heart rate data (patient cases), physiological and solving parameters, and initial conditions were input to the model. Each equation was run for multiple time iterations to obtain the outputs.

All MATLAB codes used to solve the model equations can be found at GitHub: `https://github.com/Cardiovascular-Modeling-Laboratory/OSAModel.git`.

Equation S4 was implemented into the MATLAB code as:

$$C_{pa,O_2T}\left(i+\text{floor}\left(\frac{t_{cycle}(i)}{dt(i)}\right)-1\right)=C_{pv,O_2T}(i-1)-\frac{MR_{O_2}}{Q_s(i-1)} \tag{S22}$$

$$dt(i)=\frac{t_{cycle}(i)\cdot dt(1)}{t_{cycle}(1)} \tag{S22a}$$

Here, the for loop index, i, was used to represent a single time iteration. For the patient cases, dt was made to vary proportionally with the heart rate to maintain the number of iterations defining the movement of the control volume through the entire systemic circulation, artery/arteriole system, and systemic capillaries. This was done to avoid any gaps in the output data arrays for C_{pa,O_2T}, C_{sa,O_2T}, and C_{sv,O_2T}, in the case of a decrease in heart rate between subsequent time iterations, or overwriting of previous data, in the case of an increase in heart rate. In Equation S22a, $dt(1)$ and $t_{cycle}(1)$ represent the initial conditions for dt and t_{cycle}, respectively. The $\text{floor}\left(\frac{t_{cycle}(i)}{dt(i)}\right)$ term in Equation S22 was used to determine the number of iterations defining the movement of the control volume through systemic circulation. Due to the variation of dt for the patient cases, this value remains constant. Similarly, Equations S5 and S3 were implemented as:

$$C_{sa,O_2T}\left(i+\text{floor}\left(\frac{t_{sa}(i)}{dt(i)}\right)\right)=C_{pv,O_2T}(i) \tag{S23}$$

and

$$C_{sv,O_2T}\left(i+\text{floor}\left(\frac{t_{sys,cap}(i)}{dt(i)}\right)\right)=C_{sa,O_2T}(i)-\frac{MR_{O_2}}{Q_s(i)} \tag{S24}$$

The $\text{floor}\left(\frac{t_{sa}(i)}{dt(i)}\right)$ and $\text{floor}\left(\frac{t_{sys,cap}(i)}{dt(i)}\right)$ terms were used to determine the number of iterations defining the movement of the control volume through systemic artery/arteriole system and systemic capillaries, respectively. These values also remain constant due to the varying dt in the patient cases. For all the simulations, Q_s, dt, $t_{sys,cap}$, t_{cycle}, and t_{sa} were implemented as constants in the code.

5.2 Pulmonary Capillary Mass Transfer

Equation S8 was implemented into the MATLAB code using the following setup:

$$C_{pc,O_2T}(1,i)=C_{pa,O_2T}(i) \tag{S25}$$

$$\begin{aligned}C_{pc,O_2T}(2:\text{end},i)&=C_{pc,O_2T}(1:\text{end}-1,i-1)\\&+\frac{k_{pc}}{Q_p(i-1)}\Big(C_{A,O_2}(i-1)-C_{pc,O_2d}(1:\text{end}-1,i-1)\Big)\end{aligned} \tag{S26}$$

The result of C_{pc,O_2T} was a matrix, where each column represented the spatial oxygen profile at a specific time point. In addition, the total oxygen concentration in the pulmonary veins was determined as:

$$C_{pv,O_2T}(i) = C_{pc,O_2T}(\text{end}, i) \tag{S27}$$

For all the simulations, Q_p was implemented as a constant in the code.

5.3 Alveolar Mass Transfer

Equations S12 and S13 for inspiration and expiration, respectively, were implemented into the MATLAB code as:

$$\begin{aligned} P_{A,O_2}(i) = {} & \frac{\Big(V_A(i) - V_A(i-1)\Big)\Big(P_{I,O_2} - P_{A,O_2}(i-1)\Big)}{V_A(i-1)} \\ & - \frac{k_l \cdot \beta_p \cdot dt(i-1)\Big(P_{A,O_2}(i-1) - P_{c,O_2}(i-1)\Big)R \cdot T}{V_A(i-1)} + P_{A,O_2}(i-1) \end{aligned} \tag{S28}$$

and

$$P_{A,O_2}(i) = -\frac{k_l \cdot \beta_p \cdot dt(i-1)\Big(P_{A,O_2}(i-1) - P_{c,O_2}(i-1)\Big)R \cdot T}{V_A(i-1)} + P_{A,O_2}(i-1) \tag{S29}$$

These equations were coded into a separate function. For all the simulations, dt was implemented as a constant in the code, but, for the OSA patient cases, it was varied according to Equation S22a.

5.4 Time Step for Simulations and Patient Cases

The initial time step ($dt(1)$) for the simulations and patient cases was chosen based on the following conditions: ensuring i) model stability over the period of study, ii) model convergence, and iii) maximal computational efficiency. Convergence was tested using the normal breathing simulation. The model was run at values of the time step lower than the selected value of 0.01 s (Table S3), and it was ensured that the solutions were reasonably close for each one. $dt(1)>0.01$ s was not chosen as the solution began to lose stability and diverge. Although the model can be run at $dt(1)<0.01$ s, computation time is significantly longer. Therefore, 0.01 s was chosen to maintain a balance between all three conditions. For the patient cases, since $dt(1)$ varies with the heart rate (Equation S22a), the initial time steps of 0.008 s for Patient 1 and 0.007 s for Patient 2 were chosen such that the maximum value would be 0.01 s:

$$\{dt(1) \text{ for patient cases}\} = \frac{0.01 \cdot HR_{\{\min\}}}{HR(1)} \tag{S30}$$

5.5 Estimation of OSA Patient Lung Volume Using Recorded Nasal Pressure

For Patient 1, the raw nasal pressure signal was normalized as:

$$P_N = P_{N,m} - \text{movmean}(P_{N,m},\ 70/dt_m) \tag{S31}$$

For Patient 2, the raw nasal pressure signal was normalized as:

$$P_N = P_{N,m} - \text{movmean}(P_{N,m},\ 50/dt_m) \tag{S32}$$

dt_m was the time step for measurement. Eq. S21 was implemented into the MATLAB code for each case to determine the time-dependent lung volume using cumulative integration:

$$V_{A,OSA}(1) = V_{End} \tag{S33}$$

$$V_{A,OSA}(2:\text{End}_i - \text{Start}_i + 1) = V_{End} + \text{cumtrapz}(dt_m,\ Q_N(\text{Start}_i + 1:\text{End}_i)) \tag{S34}$$

Start_i and End_i represented the starting and ending time elements for each breathing pattern, respectively, and were chosen to avoid the time period when CPAP was administered to the patients during the sleep studies as well as segments of time with any signal loss in recorded data due to patient movement. In addition, Start_i was chosen around a point where the nasal pressure was zero, before inspiration, which followed an ideal normal breath with similar inspiratory and expiratory flow rates. The resulting vector of $V_{A,OSA}$ for each patient was imported into the main code to serve as a column for a reference lookup table.

5.6 Lookup Tables

Interpolation using reference lookup tables was employed to determine the values of alveolar volume, heart rate (for patient data), and dissolved oxygen concentration at each time step. For the OSA simulations, variations of Equation 7 were used to construct breathing patterns. The time and alveolar volume data for each pattern was converted into a .mat file and imported into the main code. Heart rate was kept constant for the simulated cases. A similar conversion process was repeated for the patient data, which also included the variable heart rate input in the .mat file. The data extracted from the .mat files served as reference lookup tables in the main code. Interpolation for alveolar volume and heart rate was carried out using:

$$V_A(i) = \text{interp1}(\text{t}_{\text{data}}, \text{V}_{\text{data}}, t(i)) \tag{S35}$$

and

$$HR(i) = \text{interp1}(\text{t}_{\text{data}}, \text{HR}_{\text{data}}, t(i)) \tag{S36}$$

t_{data} (time) represented the input column of the lookup table, while V_{data} (alveolar volume) and HR_{data} (heart rate) were the output columns.

Due to the non-linearity of Equation 3 and to minimize computational effort, a lookup table was constructed to determine the dissolved oxygen concentration at each spatial position. Equation 3 was used to calculate the hemoglobin oxygen saturation for a range of dissolved oxygen concentrations. To ensure accuracy, the dissolved oxygen column in the table ranged from 0 to 2×10^{-6} mol/mL with increments of 2.5×10^{-10} mol/mL. These values were then used with Equation 2 to determine the corresponding total oxygen concentrations. This allowed the use of interpolation:

$$C_{j,O_2d}(i) = \text{interp1}(\text{CT}, \text{Cd}, C_{j,O_2T}(i)) \tag{S37}$$

CT (total oxygen concentration) represented the input column of the lookup table, while Cd (dissolved oxygen concentration) was the output column. j in Equation S37 defines the spatial location. j = pa, pc, pv, sa, and sv for the pulmonary arteries, pulmonary capillary compartment, pulmonary veins, systemic arteries, and systemic veins, respectively.

6 MODEL OUTPUTS

6.1 Comparison of Normal Breathing Results with Expected Physiological Values

Expected physiological values for the dissolved oxygen concentration in the blood vessels are not commonly found in literature. Therefore, after converting the wide range of blood oxygen partial pressures in the systemic vessels (Guyton and Hall (2000); Ortiz-Prado et al. (2019); van Faassen et al. (2009)) using β_p (Table S2), values of 119-140 μM and 38-63 μM were obtained for the systemic arteries and veins, respectively. The average values predicted by the model (Figure 1) for the systemic arteries and veins fall within these ranges. It is, however, important to note that the dissolved oxygen is sensitive to the choice of solubility factor.

6.2 Calculation of Percent Decreases for OSA Simulations

Table S5: Percent Decreases for OSA Simulations

	**Percent Decrease (%) in Variable**				
**Simulation**	S_{sa,O_2}	S_{sv,O_2}	C_{sa,O_2d}	C_{sv,O_2d}	Mass Transfer
**Sim. 1**	4.0	5.7	30	7.6	7.0
**Sim. 2**	4.0	5.6	29	7.5	1.2
**Sim. 3**	4.9	6.9	33	9.0	13
**Sim. 4**	5.7	8.0	35	10	19
**Sim. 5**	1.8	2.6	18	3.6	6.0
**Sim. 6**	1.7	2.5	17	3.5	7.2
**Sim. 7**	11	15	47	18	21
**Sim. 8**	5.4	7.5	34	9.8	20
**Sim. 9**	3.0	4.4	25	6.0	9.4
**Severe OSA**	8.0	11	41	14	8.3

Percent decreases in saturation and dissolved oxygen concentration for each simulation were calculated by comparing the average normal values to the absolute minimum points over the time of study:

$$\{\%\text{ decrease in } S_{j,O_2}\} = \left(\frac{S_{j,O_2\{\text{avg,norm}\}} - S_{j,O_2\{\text{min}\}}}{S_{j,O_2\{\text{avg,norm}\}}}\right) \cdot 100 \tag{S38}$$

$$S_{j,O_2\{\text{avg,norm}\}} = \frac{S_{j,O_2\{\text{max,norm}\}} + S_{j,O_2\{\text{min,norm}\}}}{2} \tag{S38a}$$

$$\{\%\text{ decrease in } C_{j,O_2d}\} = \left(\frac{C_{j,O_2d\{\text{avg,norm}\}} - C_{j,O_2d\{\text{min}\}}}{C_{j,O_2d\{\text{avg,norm}\}}}\right) \cdot 100 \tag{S39}$$

$$C_{j,O_2d\{\text{avg,norm}\}} = \frac{C_{j,O_2d\{\text{max,norm}\}} + C_{j,O_2d\{\text{min,norm}\}}}{2} \tag{S39a}$$

j = sa and sv for the systemic arteries and veins, respectively.

The percent decrease in mass transfer for each simulation was approximated by comparing the average difference in systemic arterial and venous dissolved oxygen for the normal condition to a temporally

averaged difference over the simulated OSA pattern:

$$\{\text{\% decrease in mass transfer}\} = \left(\frac{\Delta \mathrm{C}_{\mathrm{d,norm}} - \Delta \mathrm{C}_{\mathrm{d,OSA}}}{\Delta \mathrm{C}_{\mathrm{d,norm}}}\right) \cdot 100 \tag{S40}$$

$$\Delta \mathrm{C}_{\mathrm{d,norm}} = C_{sa,O_2d\{\text{avg,norm}\}} - C_{sv,O_2d\{\text{avg,norm}\}} \tag{S40a}$$

$$\Delta \mathrm{C}_{\mathrm{d,OSA}} = \frac{1}{t_{\mathrm{OSA}}} \int_{360}^{t_{\mathrm{total}}} \left(C_{sa,O_2d}(t) - C_{sv,O_2d}(t)\right) \mathrm{d}t \tag{S40b}$$

$$t_{\mathrm{OSA}} = t_{\mathrm{total}} - 360 \text{ s} \tag{S40c}$$

t_{OSA} is the duration of the simulated OSA breathing pattern, while t_{total} is the total time for breathing data in each case, both in seconds.

6.3 Calculation of Proposed Burden Scores

Table S6: Area Data for Proposed Burden Scores

Patient	Event Sequence	Total Sequence	
	Area Deviation from Wake Sequence (M·s)	Area Deviation from Wake Sequence (M·s)	Area Deviation from Threshold during Hypoxia (M·s)
1	0.006	0.146	0.129
2	0.009	0.024	0.023

The average arterial dissolved oxygen for wakefulness was determined as:

$$C_{sa,O_2d\{\text{avg,wake}\}} = \frac{1}{n} \cdot \sum_{k=1}^{n} C_{sa,O_2d\{\text{avg,wake},k\}} \tag{S41}$$

$$C_{sa,O_2d\{\text{avg,wake},k\}} = \frac{\int_{t_{\{k,\mathrm{s}\}}}^{t_{\{k,\mathrm{e}\}}} C_{sa,O_2d}(t)\ \mathrm{d}t}{t_{\{k,\mathrm{e}\}} - t_{\{k,\mathrm{s}\}}} \tag{S41a}$$

In Equation S41, n was defined as the total number of wake sequences considered (n = 3 for the current analysis). The subscripts s and e in Equation S41a represent the start and end times for each wake sequence, respectively. The proposed hypoxia burden score was determined as:

$$\{\text{Proposed hypoxia burden score}\} = \frac{\int_{t_1}^{t_2} \left(C_{sa,O_2d\{\text{avg,wake}\}} - C_{sa,O_2d}(t)\right) \mathrm{d}t}{t_2 - t_1} \tag{S42}$$

In Equation S42, t_1 represents the start time for the analyzed sequence (t_1 = 360 s for the total sequence), and t_2 represents the end time for the analyzed sequence. For the proposed burden score of the hypoxic period over the total patient sequence, the upper threshold was calculated as:

$$C_{sa,O_2d\{\text{hypoxia}\}} = C_{sa,O_2d\{\text{avg,wake}\}} - C_{sa,O_2d\{\text{std,wake}\}} \tag{S43}$$

$C_{sa,O_2d\{\text{std,wake}\}}$ represents the standard deviation of the arterial dissolved oxygen during wakefulness.

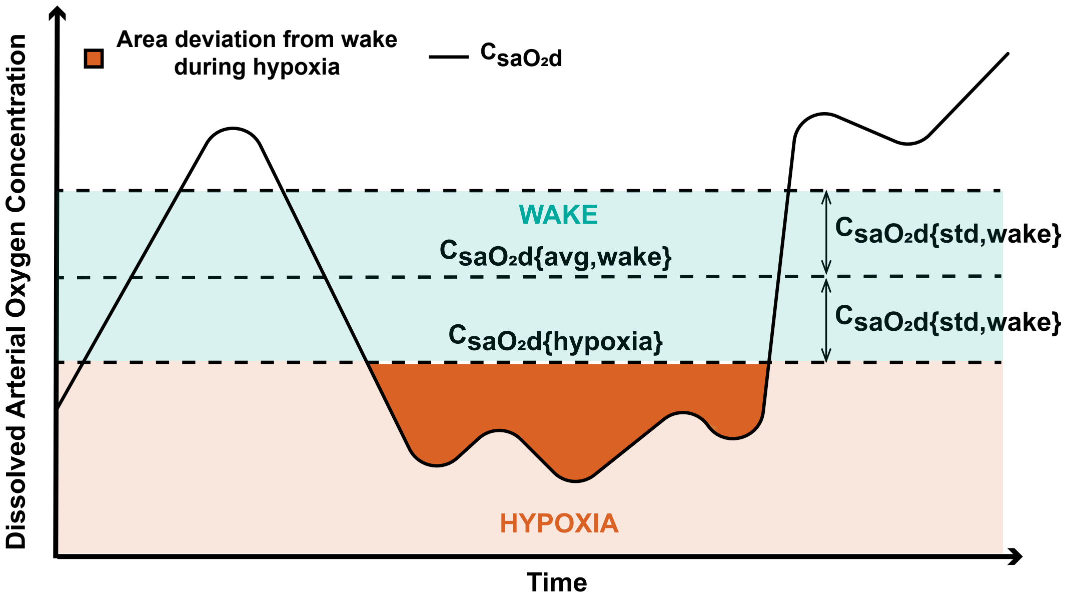

Figure S10. Schematic of area used to calculate proposed burden score for the hypoxic periods over the total sequences.

The proposed burden score for hypoxic period during the total sequence was calculated using the area deviation of the dissolved arterial oxygen from $C_{sa,O_2d\{\text{hypoxia}\}}$ (Figure S10). To determine the score, this area value was normalized by the total time for hypoxia.

6.4 Extended Data Set for OSA Patient Sleep Studies

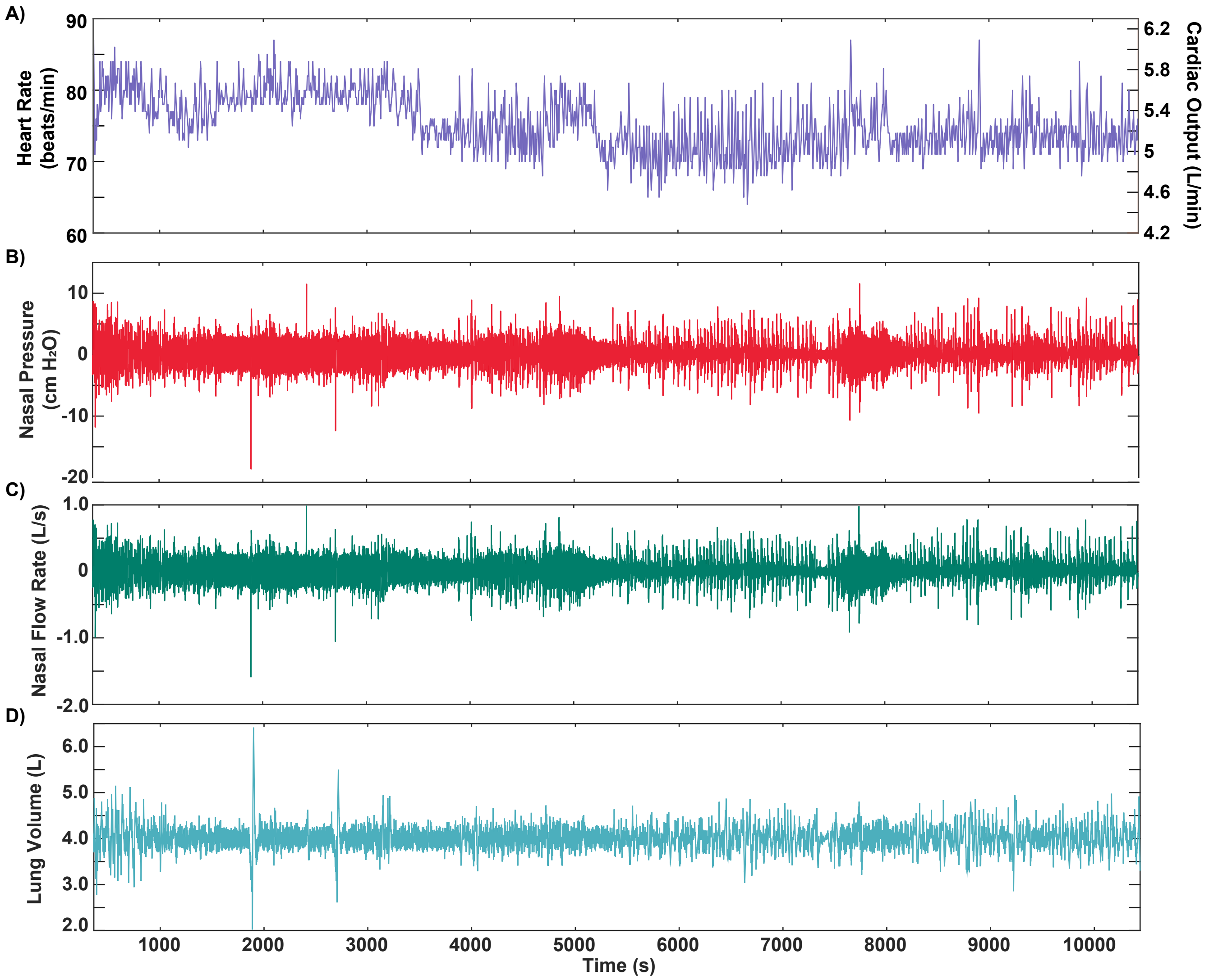

Figure S11. Patient 1 inputs to model over multi-hour portion of sleep study. A) Recorded heart rate and cardiac output. B) Raw nasal pressure. C) Nasal flow rate obtained from conversion of nasal pressure. D) Lung volume obtained from integration of nasal flow rate.

To evaluate the clinical applicability of the model over longer time periods, data from 3-hour and 2-hour portions of baseline polysomnographies, without any CPAP administration, was fed to the model for Patient 1 and 2, respectively. The heart rate and lung volume were used as direct inputs (Figures S11 and S13), while hemoglobin oxygen saturation and dissolved oxygen concentration in the systemic arteries and veins were output from the model (Figures S12 and S14).

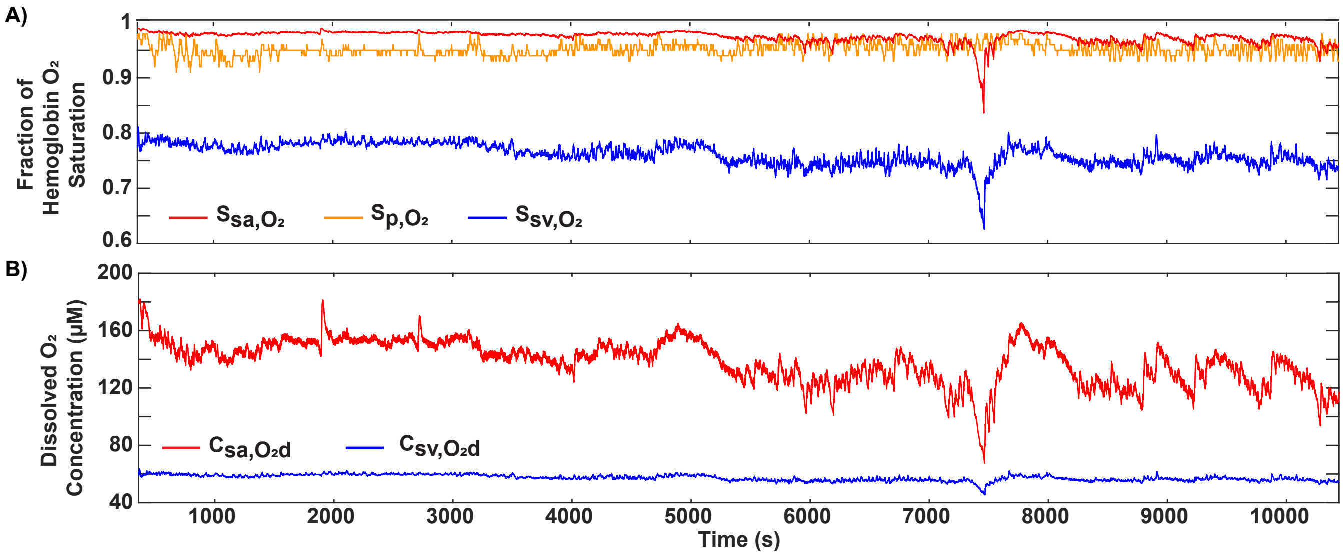

Figure S12. Patient 1 outputs from model over multi-hour portion of sleep study. A) Model output hemoglobin oxygen saturation in systemic arteries and veins, along with recorded pulse oximeter data (S_{p,O_2}). B) Model output dissolved oxygen concentration in systemic arteries and veins.

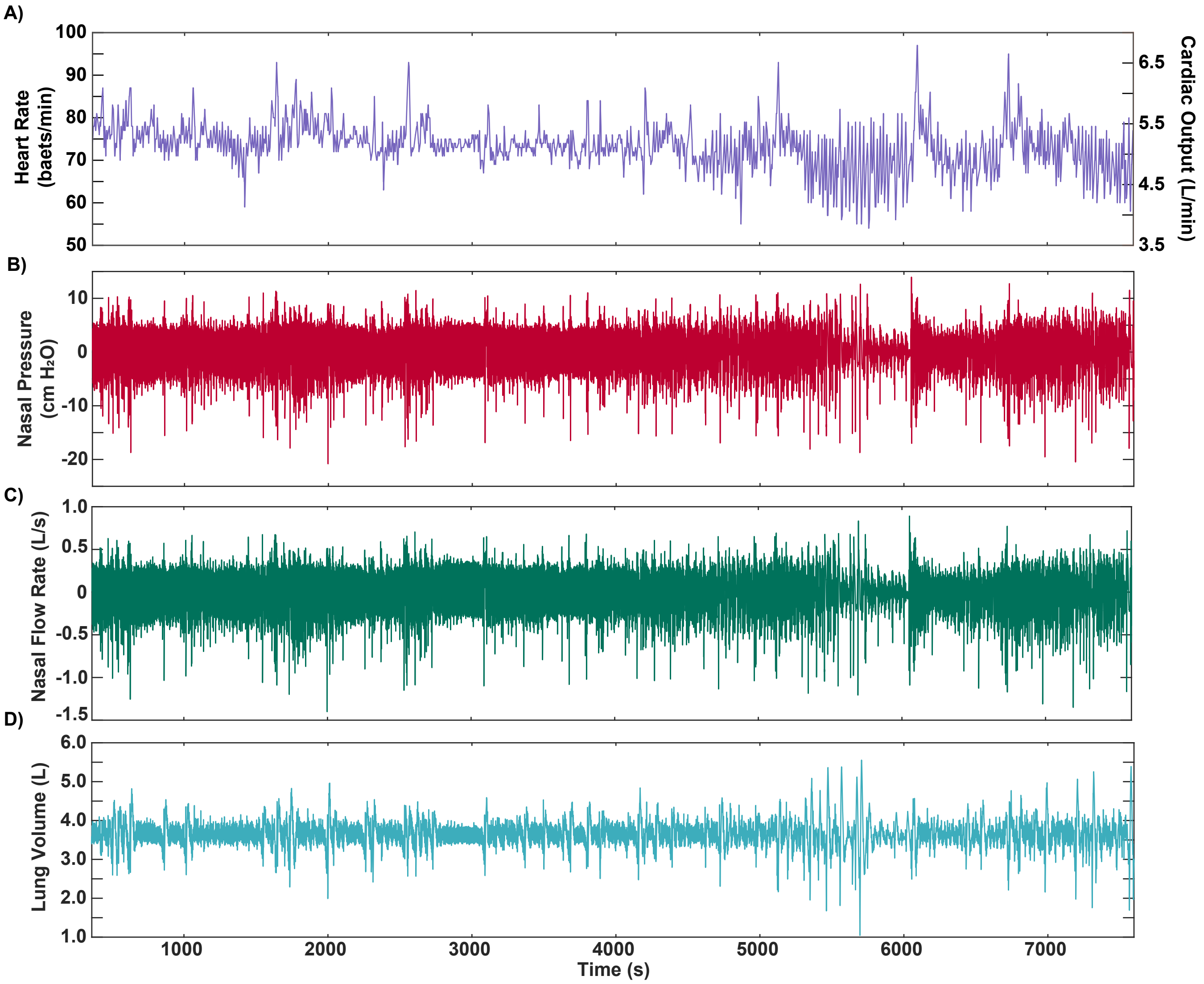

Figure S13. Patient 2 inputs to model over multi-hour portion of sleep study. A) Recorded heart rate and cardiac output.B) Raw nasal pressure. C) Nasal flow rate obtained from conversion of nasal pressure. D) Lung volume obtained from integration of nasal flow rate.

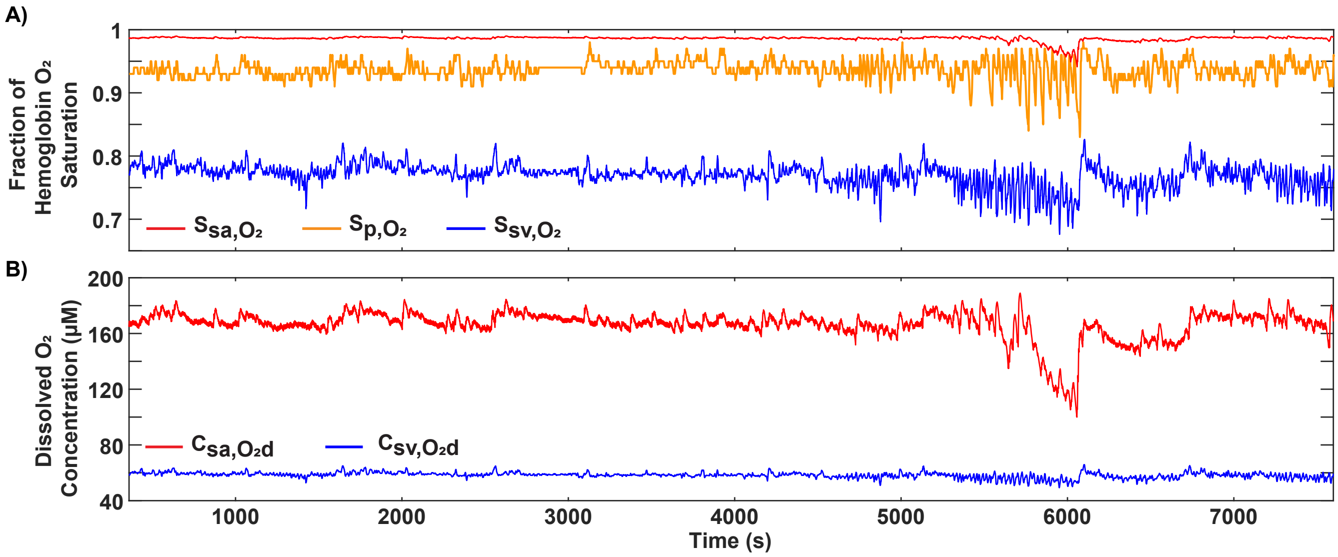

Figure S14. Patient 2 outputs from model over multi-hour portion of sleep study. A) Model output hemoglobin oxygen saturation in systemic arteries and veins, along with recorded pulse oximeter data (S_{p,O_2}). B) Model output dissolved oxygen concentration in systemic arteries and veins.

6.5 Statistical Analysis

Table S7: Data for Statistical Analysis

Variable	Overall Average	Event Series Averages
S_{sa,O_2}	0.975	0.964
		0.971
		0.957
		0.956
S_{sv,O_2}	0.762	0.740
		0.744
		0.735
		0.738
$\Delta\mathrm{C_d}$ (μM)	80.7	64.8
		72.7
		57.7
		56.8

The differences for the one-sample t-tests were calculated by subtracting the overall average of each variable from averages of the same variable over identified event series intervals:

$$\left\{\text{Difference in } S_{j.O_2}\right\} = S_{j,O_2\{\text{avg,event}\}} - S_{j,O_2\{\text{avg}\}} \tag{S44}$$

$$S_{j,O_2\{\text{avg}\}} = \frac{1}{t_{\text{study}}} \int_{360}^{t_{\text{total}}} S_{j,O_2}(t)\ \mathrm{d}t \tag{S44a}$$

$$t_{\text{study}} = t_{\text{total}} - 360\ \text{s} \tag{S44b}$$

$$S_{j,O_2\{\text{avg,event}\}} = \frac{1}{t_{\text{event}}} \int_{t_{\text{start}}}^{t_{\text{end}}} S_{j,O_2}(t)\ \mathrm{d}t \tag{S44c}$$

$$t_{\text{event}} = t_{\text{end}} - t_{\text{start}}, \quad t_{\text{start}} = \{\text{Event series start}\}, \quad t_{\text{end}} = \{\text{Event series end}\} \tag{S44d}$$

j = sa and sv for systemic arteries and veins, respectively.

$$\{\text{Difference in } \Delta\mathrm{C_d}\} = \Delta\mathrm{C}_{\mathrm{d}\{\text{avg,event}\}} - \Delta\mathrm{C}_{\mathrm{d}\{\text{avg}\}} \tag{S45}$$

$$\Delta\mathrm{C}_{\mathrm{d}\{\text{avg}\}} = \frac{1}{t_{\text{study}}} \int_{360}^{t_{\text{total}}} \left(C_{sa,O_2d}(t) - C_{sv,O_2d}(t)\right)\ \mathrm{d}t \tag{S45a}$$

$$\Delta\mathrm{C}_{\mathrm{d}\{\text{avg,event}\}} = \frac{1}{t_{\text{event}}} \int_{t_{\text{start}}}^{t_{\text{end}}} \left(C_{sa,O_2d}(t) - C_{sv,O_2d}(t)\right)\ \mathrm{d}t \tag{S45b}$$

REFERENCES

[Dataset] (2014). NIH-NHLBI ARDS Network Tools. `http://www.ardsnet.org/tools.shtml`. Accessed 2023-01-27

Abdeyrim, A., Zhang, Y., Li, N., Zhao, M., Wang, Y., Yao, X., et al. (2015). Impact of obstructive sleep apnea on lung volumes and mechanical properties of the respiratory system in overweight and obese individuals. *BMC Pulmonary Medicine* 15, 76. doi:10.1186/s12890-015-0063-6

Aird, W. C. (2005). Spatial and temporal dynamics of the endothelium. *Journal of Thrombosis and Haemostasis* 3, 1392–1406. doi:10.1111/j.1538-7836.2005.01328.x

Ben-Tal, A. (2006). Simplified models for gas exchange in the human lungs. *Journal of Theoretical Biology* 238, 474–495. doi:10.1016/j.jtbi.2005.06.005

Boron, W. F. and Boulpaep, E. L. (2009). *Medical Physiology: A Cellular and Molecular Approach* (Philadelphia, PA: Saunders Elsevier), 2nd edn.

Bruss, Z. S. and Raja, A. (2022). *Physiology, Stroke Volume* (Treasure Island, FL: StatPearls Publishing)

Cheng, L. and Khoo, M. C. K. (2012). Modeling the autonomic and metabolic effects of obstructive sleep apnea: A simulation study. *Frontiers in Physiology* 2. doi:10.3389/fphys.2011.00111

Dash, R. K. and Bassingthwaighte, J. B. (2010). Erratum to: Blood hbo2 and hbco2 dissociation curves at varied o2, co2, ph, 2,3-dpg and temperature levels. *Annals of Biomedical Engineering* 38, 1683–1701. doi:10.1007/s10439-010-9948-y

Foucquier, A., Filoche, M., Moreira, A., Andrade, J., Arbia, G., and Sapoval, B. (2013). A first principles calculation of the oxygen uptake in the human pulmonary acinus at maximal exercise. *Respiratory Physiology & Neurobiology* 185, 625–638. doi:10.1016/j.resp.2012.10.013

Frank, A. O., Chuong, C. J. C., and Johnson, R. L. (1997). A finite-element model of oxygen diffusion in the pulmonary capillaries. *Journal of Applied Physiology* 82, 2036–2044. doi:10.1152/jappl.1997.82.6.2036

Guyton, A. C. and Hall, J. E. (2000). *Textbook of Medical Physiology* (Philadelphia: W. B. Saunders), 10 edn.

Hsia, C. C., Hyde, D. M., and Weibel, E. R. (2016). Lung structure and the intrinsic challenges of gas exchange. *Comprehensive Physiology* , 827–895doi:10.1002/cphy.c150028

MacIntyre, N. R. (2014). Tissue hypoxia: Implications for the respiratory clinician. *Respiratory Care* 59, 1590–1596. doi:10.4187/respcare.03357

Ortiz-Prado, E., Dunn, J. F., Vasconez, J., Castillo, D., and Viscor, G. (2019). Partial pressure of oxygen in the human body: a general review. *American Journal of Blood Research* 9, 1–14

Popel, A. S. (1989). Theory of oxygen transport to tissue. *Critical Reviews in Biomedical Engineering* 17, 257–321

Reynolds, A., Ermentrout, G. B., and Clermont, G. (2010). A mathematical model of pulmonary gas exchange under inflammatory stress. *Journal of Theoretical Biology* 264, 161–173. doi:10.1016/j.jtbi.2010.01.011

van Faassen, E. E., Bahrami, S., Feelisch, M., Hogg, N., Kelm, M., Kim-Shapiro, D. B., et al. (2009). Nitrite as regulator of hypoxic signaling in mammalian physiology. *Medicinal Research Reviews* 29, 683–741. doi:10.1002/med.20151
